# Loss of Brg1 prevents the progression of pulmonary hypertension by inhibiting Nrf2 expression

**DOI:** 10.1038/s41598-026-55301-6

**Published:** 2026-06-03

**Authors:** Wen-Di Jiang, Mei-Yang Xu, Zi-yi Chen, Qing Chen, Guo-Qing Lu, Zheng-Yu Sun, Jia-Yi Guo, Lei Wang, Ying Qian, Li Mu, Yu-Hang Wang, Ning-ning Zhang, Hong-Ju Wang, Bi Tang, Pin-Fang Kang, Wen-Juan Wu

**Affiliations:** 1 Key Laboratory of Cancer Research and Clinical Laboratory Diagnosis, Bengbu Medical University, Bengbu, 233000 Anhui People’s Republic of China; 2 Department of Biochemistry and Molecular Biology, School of Laboratory Medicine, Bengbu Medical University, Bengbu, 233000 Anhui People’s Republic of China; 3 Department of Cardiovascular Medicine, The First Affiliated Hospital of Bengbu Medical University, Bengbu, 233000 Anhui People’s Republic of China; 4 School of Laboratory Medicine, Bengbu Medical University, Bengbu, 233000 Anhui People’s Republic of China; 5 School of Clinical Medicine of Bengbu Medical University, Bengbu, 233000 Anhui People’s Republic of China; 6 Department of Cardiovascular Disease, The First Affiliated Hospital of Bengbu Medical College, 287 Chang Huai Avenue, Bengbu, 233004 People’s Republic of China; 7 Department of School of Laboratory Medicine, 2600 Dong Hai Avenue, Bengbu, 233030 People’s Republic of China

**Keywords:** Brahma-related gene 1, Oxidative stress, Cell proliferation, Nuclear factor erythroid-2 related factor 2, Pulmonary artery hypertension, Molecular biology, Physiology, Systems biology, Cardiology, Medical research

## Abstract

Brahma-related gene 1 (Brg1) is a major factor in regulation of chromatin remodeling and is involved in different cellular processes, including cell proliferation, apoptosis, and differentiation. However, the role of Brg1 in pulmonary artery hypertension (PAH) has not been elucidated. This study aims to assess the role of Brg1 in PAH using hypoxia-induced PAH rat model, Monocrotaline(MCT)-induced PAH rat model, and hypoxia-induced arterial smooth muscle cell model. We found that Brg1 expression was highly expressed in hypoxia-induced PAH rat model and MCT-induced PAH rat model. Moreover, knockdown of Brg1 by injection of adenoviral Brg1 shRNA inhibited Nrf2 expression and its downstream molecular HO-1, attenuated apoptosis resistance, and ameliorated PAH progression in PAH rats. Meanwhile, knockdown of Brg1 suppressed cell proliferation through downregulating the expression of mTOR/P70S6K signaling pathway in vitro and in vivo. In addition, the Nrf2 inhibitor Brusatol treatment significantly inhibited cell proliferation, whereas abrogated by loss of Brg1. In summary, these results demonstrated that loss of Brg1 inhibited PAH progression by downregulating Nrf2 to attenuate cell proliferation and enhance apoptosis, which may provide a new therapeutic target to improve cardiopulmonary function in PAH patients.

**Supplementary Information:**

The online version contains supplementary material available at 10.1038/s41598-026-55301-6.

## Introduction

 Pulmonary artery hypertension (PAH) is a fatal cardiovascular disease characterized by elevated pulmonary artery pressure and right heart failure. PAH currently affects more than 100 million people globally, with a 5-year survival rate of only 50%, and has been described as the “cancer” of cardiovascular diseases due to its extremely poor prognosis^1^. Pulmonary artery smooth muscle cells (PASMCs) are a major component of the intima-media of the pulmonary artery vasculature and play an important role in pulmonary vascular remodeling^2^. Proliferation of PASMCs as well as injury to pulmonary vascular endothelial cells are the main pathologic changes in PAH. Under normal physiological conditions, the proliferation and apoptosis of PASMCs are maintained in a dynamic equilibrium. When the proliferation of PASMCs is excessive or apoptosis is inhibited, it will lead to the thickening of the smooth muscle layer in the intima-media of the pulmonary vascular wall and pulmonary vascular remodeling, which will ultimately promote the progression of right heart failure^3^. Therefore, the study of inhibiting the proliferation of PASMCs and inducing apoptosis is critical to the recovery of pulmonary vascular injury in pulmonary hypertension.

Chromatin remodeling is a type of cellular epigenetic modification. ATP-dependent chromatin remodeling complex SWI/SNF (Switch defective /Sucrose Non-Fermentable) is divided into BAF (BRG1/BRM-associated factors) and PBAF (Polybromo-associated BAF) isoforms. Brahma-related gene 1 (Brg1) is one of the important chromatin remodeling factors and is the core ATPase of BAF, whose presence determines the basic activity of the complex^4^. It relies on ATP hydrolysis to generate energy, contributes to histone and DNA depolymerization, maintains complex stability by binding to other subunits through the C-terminal domain. It is also target-specific, able to localize to specific genomic regions dependent on transcription factors or epitopes and alter chromatin morphology in a context-dependent manner. Brg1 plays an important role in the regulation of DNA replication, repair, recombination, transcription and expression, and signaling pathways^5–7^. Previous studies have shown that Brg1 plays an important role in the early dysfunction of aortic smooth muscle cells, converting the smooth muscle phenotype and participating in the development of vascular calcification^8^. Emerging evidence suggests that Brg1 regulates endothelial cell function and cardiac hypertrophy^9,10^. In addition, Brg1 also plays an important role in neural stem cells and glial cells^11^, cardiomyocytes^12^, hepatocellular carcinoma^13^, prostate cancer cell proliferation and differentiation^14^, and hair follicle cell regeneration and repair^15^. However, the role of Brg1 in pulmonary vascular smooth muscle cell proliferation, migration, and repair after PAH is unknown. Nuclear factor erythroid-2 related factor 2 (Nrf2) is a basic leucine zipper transcription factor of the cap’n’collar (CNC) family, which is mainly involved in regulating the antioxidant defense system in vivo and modulates the expression of antioxidant factors^16^. When the organism is stimulated by hypoxia and other stimuli, Nrf2 translocates into the cell nucleus and forms a heterodimer that can bind to antioxidant response element (ARE) and initiate the transcription of downstream genes, realizing a variety of functions such as anti-oxidative stress, anti-inflammatory damage, and anti-apoptosis, thus stabilizing the cellular environment^17^. Traditionally, Nrf2 activation has been considered protective against oxidative injury and vascular remodeling^18^. However, some evidence indicates that persistent or excessive activation of Nrf2 may induce abnormal metabolic adaptation, enhanced mitochondrial activity, and apoptosis resistance, which paradoxically exacerbate vascular remodeling under chronic hypoxia. Furthermore, previous studies have shown that overexpression of Brg1 can increase Nrf2 expression, promoting its nuclear translocation and transcriptional activation^19^. However, the mechanism by which Brg1 mediates the role of Nrf2 in pulmonary hypertension remains to be elucidated.

Oxidative stress is an imbalance in redox homeostasis caused by excessive production of highly reactive molecules such as reactive oxygen radicals^20^. An imbalance between the oxidative and antioxidant systems in the lungs induces lipid peroxidation and cellular damage, which leads to endothelial dysfunction in the pulmonary vasculature, smooth muscle proliferation and migration, and the formation of vascular remodeling^21,22^. Multiple types of pulmonary hypertension occur in association with oxidative stress. In the present study, we evaluated the role of Brg1 in the development of experimental pulmonary hypertension induced by chronic hypoxia (Hypoxia) and Monocrotaline (MCT) in rats. The expression and function of Brg1 in PASMCs were also investigated. In conclusion, we found that Brg1 is involved in PAH pathological phenotypes and these abnormalities lead to PA remodeling. Therefore, Brg1 may be a novel therapeutic target for the treatment of PAH.

## Materials and methods

### Animals

The rats were divided into 3 groups: control group (Control), Hypoxia+Brg1 shRNA adeno-associated virus empty vector group (Hypoxia), and Hypoxia+ Brg1 shRNA adeno-associated virus (WZ Biosciences Inc, China) group (Hypoxia +shBrg1). The transfected virus titer was 1 × 10^13^ vg/mL, injected via tail vein in a dose of 200 µL (2 × 10^12^ vg) per animal, and kept for 2 weeks and then placed in a hypoxic environment (10% atmospheric hypoxia) for 4 weeks to establish a model of hypoxia (PAH). The MCT model was also established by a single subcutaneous injection of 60 mg/kg MCT (Sigma, USA, C2401) in SD rats 2 weeks after transfection with adeno-associated virus, and the SD rats were divided into three groups: control group (Control), MCT + Brg1 shRNA adeno-associated viral empty vector (MCT), and MCT+Brg1 shRNA adeno-associated viral group (MCT+shBrg1).

### Echocardiography

After modeling, the rats in each group were placed in a small animal anesthesia box measuring 240 × 120 × 180 mm (RWD, China) and anesthetized with isoflurane (Baxter International, USA) at a concentration of 3%, and the flow rate of the anesthesia box was adjusted to 2.5 L/min for anesthesia. After being deeply anesthetized, they were immediately taken out of the anesthesia box and placed on the rat carrier plate, and the anesthesia was maintained by passing isoflurane through the mouth and nose, and the flow rate was adjusted according to the body weight of the rats. Cardiac ultrasound localization of the pulmonary arteries and right ventricle was performed using the S250 probe of the small animal ultrasound machine Vevo 2100 (VisualSonics, Canada), and right ventricular free wall thickness (RVFW), pulmonary acceleration time (PAT), and pulmonary ejection time (PET) were recorded and measured in each group.

### Hemodynamic assessment and determination of right ventricular hypertrophy index (RVHI)

After anesthetizing the rats with 2% pentobarbital sodium, an incision was made outside the midline of the right cervical clavicle to expose the right jugular vein. The right external jugular vein was gradually freed using curved forceps, and a PE50 model polyethylene catheter (Smiths Medical, UK, 187241) was inserted into the right atrium and right ventricle from the right common carotid vein. The pressure waveforms were recorded with a biopressure transducer, and the pressure waveforms on the monitor represented the right ventricular systolic pressure (RVSP). After hemodynamic assessment, the whole heart was rapidly dissected and the right ventricle (RV), left ventricle (LV), and septum (S) were weighed. The right ventricular hypertrophy index was then calculated as the weight ratio of the right ventricle to the LV plus the septum.

### Arterial ring studies

Rats were anesthetized by intraperitoneal injection of 2% sodium pentobarbital at a dose of 150 mg/kg. The heart and lungs were then removed intact, and the pulmonary artery was dissected from the lungs. Subsequently, a 2–3 mm vascular ring was cut out and suspended between two tungsten wires in a tissue bath. Initial pre-tension was set at 1.0 g and subsequently adjusted to 1.5 g and equilibrated for 30 min to stabilize the baseline. The solution was continuously ventilated with a mixture of 95% O_2_ and 5% CO_2_, maintained at 37 °C, and recorded with a biopressure sensor. The vasoconstrictive effect of each group was tested by administering 60 mmol/L KCl solution after stabilization. Reequilibration with fresh K-H solution was performed three times, followed by sequential addition of acetylcholine(Ach) solution with cumulative concentrations of 1 × 10^− 9^, 1 × 10^− 8^, 1 × 10^− 7^, 1 × 10^− 6^, 1 × 10^− 5^, and 1 × 10^− 4^ mol/L, and percent diastole was calculated. Similarly, Norepinephrine(NE) at concentrations of 1 × 10^− 10^, 1 × 10^− 9^, 1 × 10^− 8^, 1 × 10^− 7^, 1 × 10^− 6^, and 1 × 10^− 5^ mol/L was used to induce contraction of vascular rings in each group, and line graphs were plotted on the basis of contraction force.

### Histopathological assay

Heart and lung tissues were fixed in 4% paraformaldehyde for 48 h, dehydrated and embedded in paraffin, and then cut into section sections with a thickness of 4 μm after the paraffin solidified. Hematoxylin eosin (HE) staining was performed to observe the morphology of heart and lung tissues. The immunohistochemical staining of lung tissue sections was stained with Brg1 antibody (Servicebio, China, GB11258). The results were observed by an electron microscope.

### Cell culture and adeno-associated virus type 9 (AAV9) transfection

Pulmonary artery smooth muscle cells (PASMCs) were isolated from three 8-week-old, male SD rats (weighing approximately 250–300 g). The animals were anesthetized and then aseptically dissected from the pulmonary artery, stripped of the intimal and extimal tissues, and the middle layer of the tissue was left chopped and placed in DMEM (Gbico, USA, 11965092) culture medium containing 10% fetal bovine serum (Gbico, USA, A5256701) for adherent culture. Cultured PASMCs were infected with Brg1 shRNA AAV9 (WZ Biosciences Inc, China) for 48 h (MOI = 100), respectively, according to the instructions. Cells were divided into three groups: control group (Control), hypoxia+Brg1 shRNA empty vector group (Hypoxia), and hypoxia+Brg1 shRNA group (Hyp+shBrg1). In this case, the hypoxic approach was to incubate the cells in a three-gas incubator (5% CO_2_, 3% O_2_) for 48 h. To investigate the effect of Nrf2 on cell proliferation, cells were treated with Brusatol (MedChemExpress, USA, HY-19543), an Nrf2 inhibitor at a concentration of 10 mM, and Curcumin (MedChemExpress, USA, HY-N0005), an Nrf2 agonist at a concentration of 20 mM, for 24 h. The cells were divided into five groups: control group (Control), hypoxia + Brg1 shRNA empty vector group (Hypoxia), hypoxia+Brg1 shRNA group (Hyp+shBrg1), hypoxia + Brg1 shRNA + Brusatol group (Hyp+shBrg1 + Brusatol), and hypoxia+Brg1 shRNA + Curcumin group (Hyp+shBrg1 + Curcumin).

### CCK-8

Cells were inoculated into 96-well plates at 3000/well, and 6 replicate wells were set up in each group, and the intervention was carried out after the cells were attached to the wall. After the intervention, 10uL of CCK-8 working solution (Beyotime, China, C0042) was added to each well, and the cells were incubated at 37 ℃ for 2 h. After CCK-8 incubation, the 96-well plate was removed and the absorbance at 450 nm was measured in an enzyme counter and recorded.

### Measurement of biomarkers of oxidative stress

Pulmonary artery smooth muscle cells were collected and lysed, isolated lung tissues were homogenized, and the supernatant was collected. MDA and SOD were detected in each group of cells using Malondialdehyde (MDA) Detection Kit (Solarbio, China, BC0025) and Superoxide Dismutase (SOD) Detection Kit (Solarbio, China, BC5165) according to the instructions.

### RNA isolation and RT-qPCR

RNA was extracted from lung tissues using an RNA extraction kit (Vazyme, China, RC112-01), and the extracted RNA was reverse transcribed to cDNA using a reverse transcription kit (Vazyme, China, R333). Fluorescence quantitative PCR was performed after subsequent addition of specific primers and amplification kits (Vazyme, China, Q711). Primer sequences were as follows: Brg1 (Forward 5’-TCTCATGCACAGAGGTCCTG -3’; Reverse 5’-TAGCCCCTTGAAAGTGATCC-3’) ; β-actin (Forward 5’-CCTGGCACCCAGCACAAT-3’; Reverse 5’-GGGCC GGACTCGTCATAC-3’).

### Immunofluorescence

Fixed lung tissue sections and treated PASMCs were perforated and membrane-broken in 0.3% Triton X-100 for 15 min at room temperature, washed with PBST, and then incubated with 1% bovine serum albumin (BSA) (Biosharp, China, BS114-250 g) for 1 h. The sections and smooth muscle cells were then incubated with primary antibody at 4 °C overnight. The next day, the sections and smooth muscle cells were rinsed three times with PBST and incubated for 2 h with secondary antibody. Cell nuclei were restained with DAPI Staining Solution (Biosharp, China, BL105A). Images were captured with a fluorescence inverted microscope and analyzed with Image J software. Primary antibodies used were: Ki67 (Cell Signaling Technology, USA, D3B5), α-SMA (Servicebio, China, GB111364). Secondary antibodies used: Cy3 (ABclonal, China, AS007), Cy5 (Servicebio, China, GB27303).

### Superoxide anion assay

The treated PASMCs and tissues were incubated with 10 µM dihydroethidium (DHE; MedChemExpress, USA, HY-D0079) at 37 °C for 30 min, and then the nuclei of the cells were restained with DAPI solution, and observed and photographed under a fluorescence microscope. The fluorescence intensity of DHE-positive cells was analyzed by Image J software.

### Flow cytometry

The Annexin V-FITC/PI double staining detection kit (Beyotime, China, C1383L) was used to detect early and late apoptosis. After the cells were added to the binding solution, Annexin V-FITC and PI were added at a ratio of 1:2 for 15 min and 5 min, respectively, to avoid light staining. Apoptotic cells were detected by flow cytometry (FACSVerse, BD, USA). FlowJo software was used to calculate the apoptotic rate of the cells.

### The terminal-deoxynucleotidyl transferase dUTP-biotin nick end-labeling (TUNEL) assay

PASMCs were stained with the In Situ Apoptosis Detection Kit (Beyotime, China, C1090). The TUNEL positive rate was observed and analyzed with Image J software.

### Western blot

The supernatant was collected after cell lysis, tissue mincing and homogenization by centrifugation, and the protein concentration in the supernatant was determined by using an enhanced BCA protein assay kit (Beyotime, China, P0010), and protein gel electrophoresis samples were prepared by adding 5×loading-buffer for western blotting up. After electrophoresis, proteins were transferred to a PVDF membrane. The membrane was closed and incubated with primary antibody at 4 °C overnight. The next day, the membrane was rinsed with tris buffer containing Tween 20 (TBST), and then the secondary antibody (Biosharp, China, BL002A/ BL003A) was incubated at room temperature for 2 h before the protein level was detected using an ECL kit (Thermo Fisher Scientific, USA, 34580). The bands were analyzed using Image J software. The primary antibody used has: Brg1 (Cell Signaling Technology, USA, D1Q7F), Nrf2 (ABclonal, China, A3577), HO-1(Proteintech, China, 2D10A5), β-actin(ABclonal, China, AC038), Lamin-B1(ABclonal, China, A11495), Bcl-2 (ABclonal, China, A19693), BAX (Cell Signaling Technology, USA, D2E11), mTOR (Cell Signaling Technology, USA, 7C10), p-mTOR (Proteintech, China, 2A12G3), P70S6K (Cell Signaling Technology, USA, E8K6T), p- P70S6K (Cell Signaling Technology, USA, 108D2).

### Statistical analysis

Statistical analysis was performed using GraphPad Prism 8 software to analyze and graph the experimental data. Independent samples t-tests were used for comparisons between two groups, and one-way analysis of variance (ANOVA) was used for comparisons between three or more groups to determine differences between groups. For experiments with small sample sizes or non-normally distributed data, we additionally performed non-parametric tests (Mann–Whitney U test or Kruskal–Wallis test) to verify the consistency of the results. *P* < 0.05 was considered statistically significant. All experimental results were confirmed by at least three independent experiments.

### Animal ethics approval and compliance statement

All animal experiments in this study were performed in accordance with the ARRIVE 2.0 guidelines and the Guide for the Care and Use of Laboratory Animals. All experimental protocols were reviewed and approved by the Medical Ethics Committee of Bengbu Medical College (Reference number: [2019] No. 094). The experiments were conducted in strict accordance with animal welfare principles, employing humane methods of anesthesia and euthanasia to minimize animal suffering and reduce the number of animals used.

## Results

### Brg1 expression is increased in PAH models

**Fig. 1:**
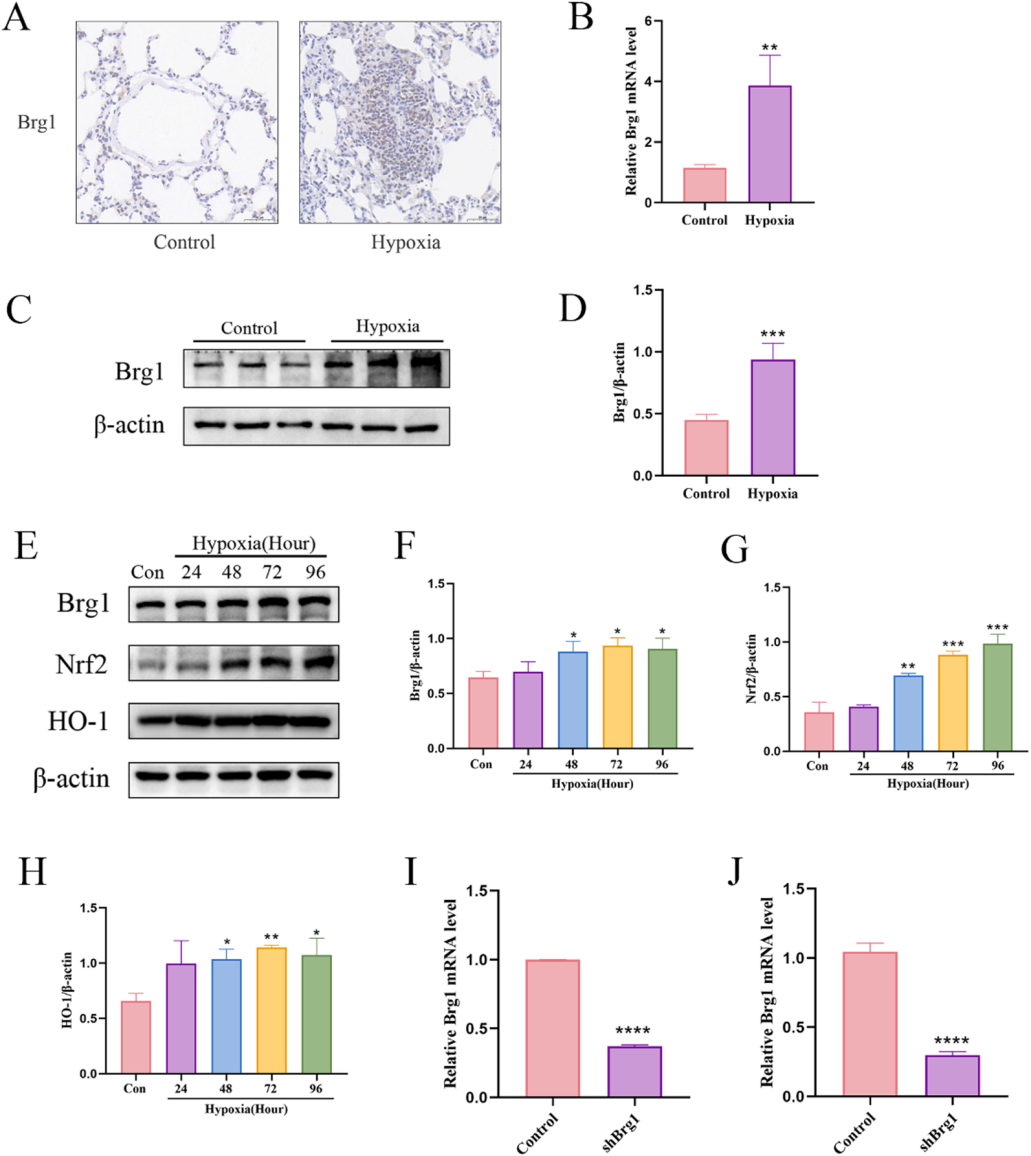
Brg1 expression was increased in PAH models. (**A**) Brg1 expression in lung tissues of PAH rats. (**B**) RT-qPCR detection of Brg1 mRNA expression in rat lung tissues. (**C**, **D**) Western blot detection of Brg1 protein expression in rat lung tissues and statistical analysis. (**E–H**) Western blot detection of Brg1, Nrf2, HO-1 protein expression in PASMCs and statistical analysis. (**I**) RT-qPCR detection of mRNA expression after knockdown of Brg1 in PASMCs. (**J**) RT-qPCR detection of mRNA expression after knockdown of Brg1 in rat lung tissues. Data was expressed as mean ± SD, *n* = 3. *P < 0.05; **P < 0.01; ***P < 0.001; ****P < 0.0001.

A PAH rat model was constructed in a hypoxic chamber. The expression of Brg1 was evaluated in rat lung tissues. The results showed that the expression of Brg1 in small pulmonary arteries of hypoxic rats was significantly higher than that of normal rats (Fig. 1A). As expected, we found that Brg1 mRNA levels as well as protein levels were significantly increased in the Hypoxia group compared with the Control group (Fig. 1B, C and D). Next, we found that in PASMCs compared to controls, Brg1 began to show a significant upward trend after 48 h of hypoxia (Fig. 1E and F). Moreover, the Nrf2 expression gradually increased in a time-dependent manner at 48 h of hypoxia (Fig. 1G). The expression of its downstream antioxidant factor HO-1 showed a significant increase at 48 h of hypoxia (Fig. 1H). Thus, we chose to mimic PAH using hypoxic PASMCs for 48 h in vitro.

We next verified the knockdown efficiency of Brg1 virus by RT-qPCR and showed that the knockdown efficiency of Brg1 adeno-associated virus in PASMCs was 63% (Fig. 1I). In animal lung tissues, the knockdown efficiency of Brg1 was 71% (Fig. 1J), indicating that Brg1 was effectively suppressed after transfection of the virus.

### Brg1 knockdown alleviated PAH progression in the lungs and right ventricle of hypoxic rats

**Fig. 2:**
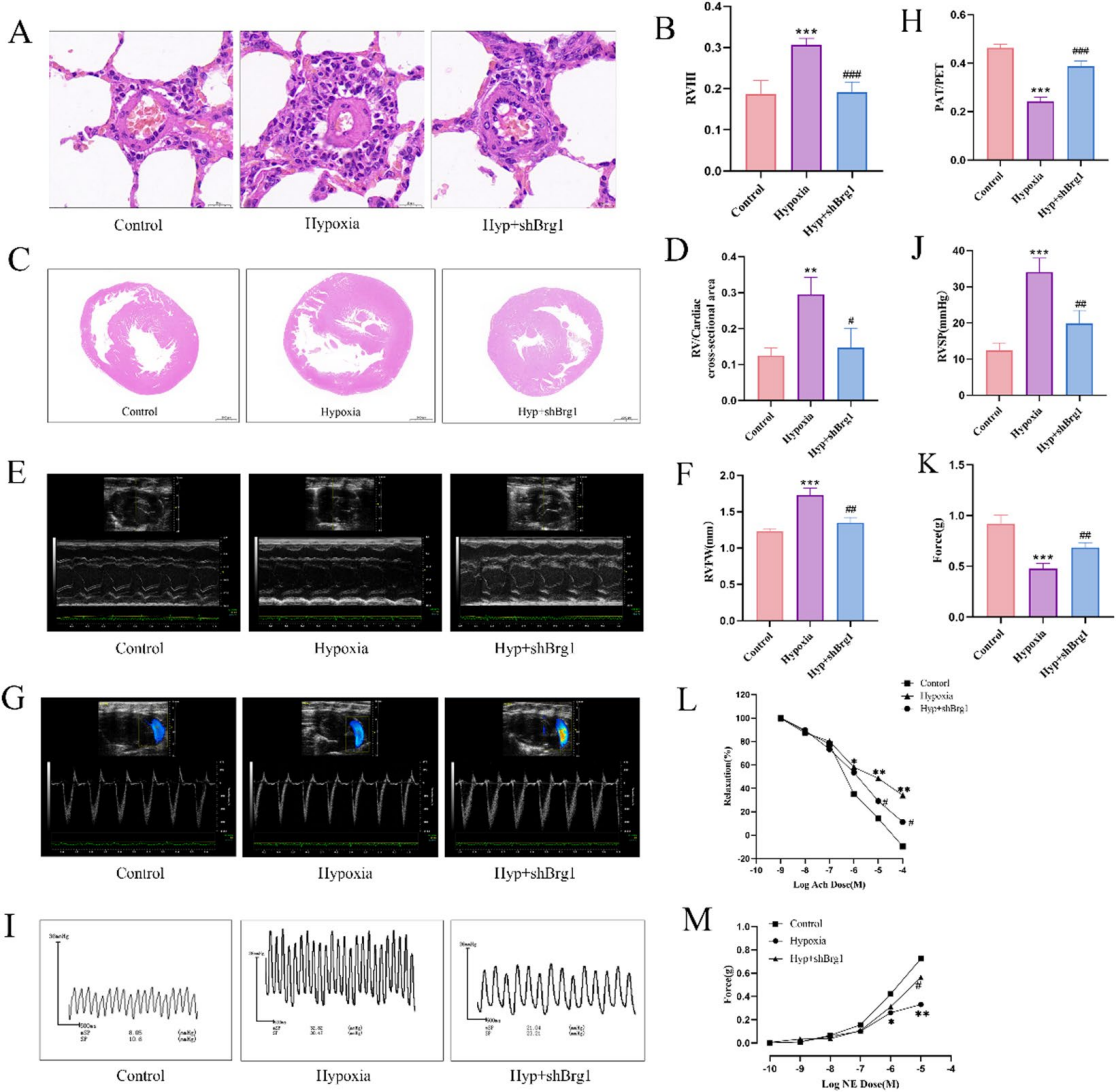
Brg1 knockdown alleviated PAH progression in the lungs and right ventricle of hypoxic rats. (**A**) HE staining results of rat lungs in Control, Hypoxia, and Hyp+shBrg1 groups. (**B**) Right ventricular hypertrophy index of rats in each group. (**C**) HE staining of cross-sections of the right ventricle of rats in various groups. (**D**) Statistical analysis of right ventricular cross-sections and chamber occupancy of rats in each group. (**E, F**) Right ventricular free wall thickness of rats in each group and graphs of statistical analysis. (**G, H**) Ratio of PAT and PET in each group of rats and graphs of statistical analysis. (**I, J**) RVSP in each group of rats and graphs of statistical analysis. (**K**) Changes in KCL-induced changes in pulmonary vasoconstriction. (**L**) Changes in Ach-induced changes in percentage of pulmonary vasodilatation. (**M**) NE-induced changes in pulmonary vasoconstriction in each group of rats. Data was expressed as mean ± SD, *n* = 3. *Compared with Control group, *P < 0.05; **P < 0.01; ***P < 0.001; ^#^Compared with Hypoxia group, ^#^*P* < 0.05; ^##^*P* < 0.01; ^###^*P* < 0.001.

To investigate the role of Brg1 in hypoxia-induced PAH in rats, we established a hypoxic PAH rat model. HE staining was used to evaluate the pathological changes of lung tissues in PAH followed by loss of Brg1. The results showed that Brg1 knockdown attenuated small pulmonary artery wall thickening (Fig. 2A). The increase in RVHI (Fig. 2B) and right ventricular cross-sectional area (Fig. 2C and D) was significantly suppressed in the Hyp+shBrg1 group compared with the Hypoxia group.

After four weeks of hypoxic intervention, our echocardiographic data showed a significant improvement in right ventricular free wall thickness (RVFW) as well as in the ratio of pulmonary artery acceleration time (PAT) to pulmonary ejection time (PET) in Brg1 knockdown rats (Fig. 2E, F, G and H). In addition, the results of right ventricular systolic pressure (Fig. 2I and J) showed that RVSP was significantly elevated in the Hypoxia group, while reduced after treatment with Brg1 adeno-associated virus, suggested that knockdown of Brg1 significantly attenuated RVSP in PAH rats.

In addition, our vascular ring results also suggested that the contractility of pulmonary arteries gradually increased with the gradual increase in NE concentration (Fig. 2M). Among them, there began to be a significant decrease in contractility in the Hypoxia group compared with the Control group at a concentration of 10^− 5^ mol/L NE, indicating a decrease in vascular tone. The vascular contractility of the Hyp+shBrg1 group was elevated compared with that of the Hypoxia group at a concentration of 10^− 5^ mol/L NE. Similarly, in the KCL-induced pulmonary artery contraction experiments (Fig. 2K), there was a significant decrease in contractility in the Hypoxia group and a significant rebound in vasoconstriction in the Hyp+shBrg1 group. The results in Ach-induced pulmonary artery diastole experiments (Fig. 2L) also showed that pulmonary vasodilatation was significantly attenuated in hypoxic PAH rats and the percentage of vasodilation was increased in the Hyp+shBrg1 group. These results suggest that loss of Brg1 improved PAH by regulating the vascular tone of pulmonary arteries.

### Brg1 knockdown attenuated oxidative stress levels and reduced apoptosis resistance in PAH of hypoxic rats

**Fig. 3:**
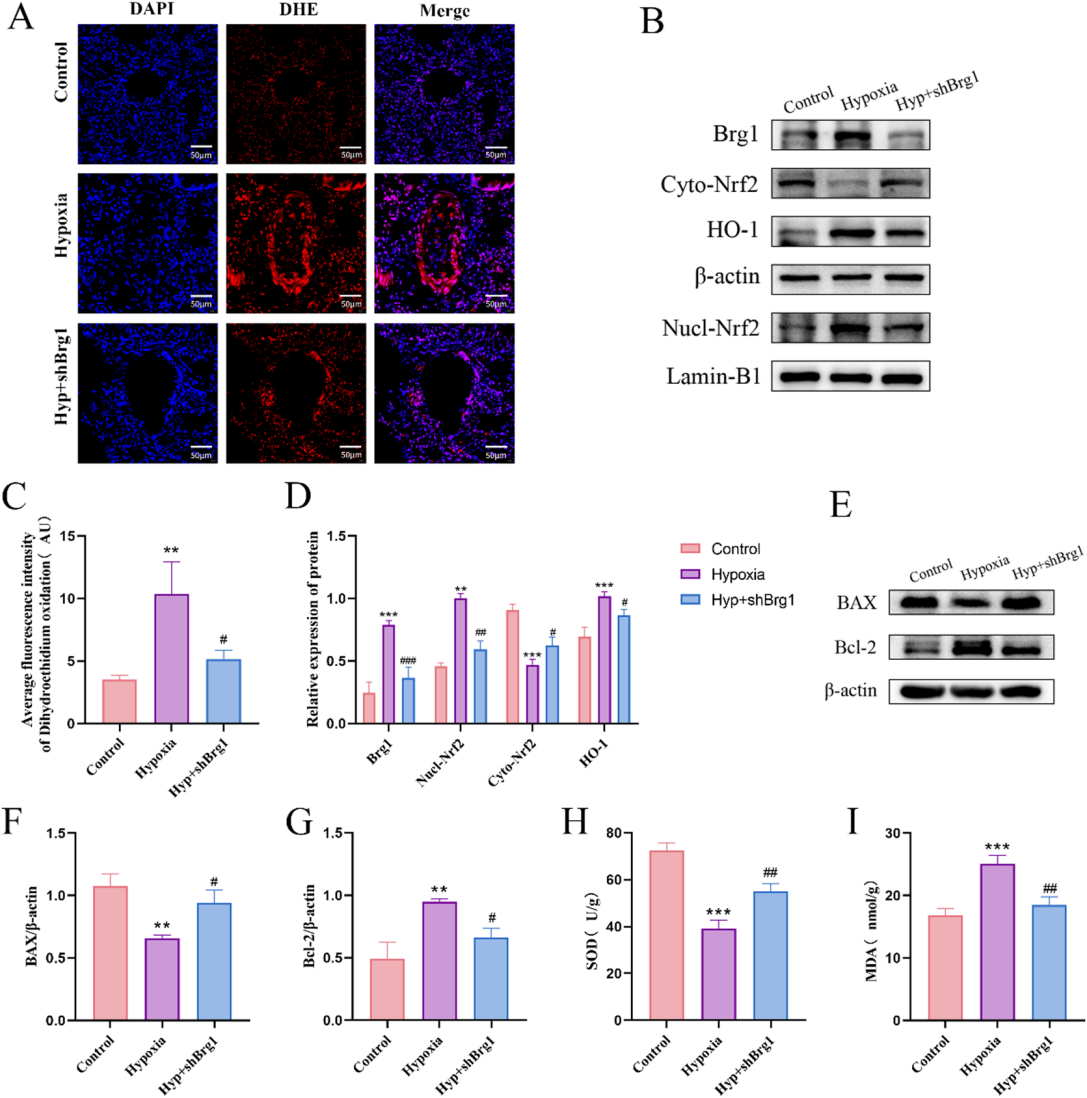
Brg1 knockdown attenuated oxidative stress levels and reduced apoptosis resistance in PAH of hypoxic rats. (**A**) Dihydroethidium (DHE) staining of pulmonary vessels was performed in the control, hypoxia and Hyp+shBrg1 groups. (**B**) Western blot detection of Brg1, Nucl-Nrf2, Cyto-Nrf2, and HO-1 expression in lung tissues. (**C**) Statistical analysis of average fluorescence intensity of DHE. (**D**) Statistical analysis of Brg1, Nucl-Nrf2, Cyto-Nrf2, and HO-1 protein expression graph. (**E**) Western blot detection of Bcl-2, BAX expression in lung tissues. (**F**) Statistical analysis of BAX2 protein expression graph. (**G**) Statistical analysis of Bcl-2 protein expression graph. (**H**) SOD content in rat lung tissues. (**I**) MDA level in rat lung tissues. Data was expressed as mean ± SD, *n* = 3. *Compared with Control group, **P < 0.01; ***P < 0.001; ^#^Compared with Hypoxia group, ^#^*P* < 0.05; ^##^*P* < 0.01; ^###^*P* < 0.001.

The changes in oxidative stress-related indices in hypoxic PAH rats after Brg1 upregulation and after treatment with Brg1 adeno-associated virus injection intervention. We found that the DHE fluorescence results showed that compared with the hypoxia group, the oxidative stress level around the small pulmonary artery was significantly increased in the hypoxia group; Knockdown of Brg1 significantly reduced oxidative stress levels in pulmonary arterioles (Fig. 3A and C). We next examined the levels of SOD and MDA, indicators related to oxidative stress, in lung tissues, and the results showed that the Hyp+shBrg1 group had lower MDA levels and higher SOD content, indicating lower levels of oxidative stress, compared with the Hypoxia group (Fig. 3H and I).

To investigate the expression of the Brg1/Nrf2/HO-1 oxidative stress signaling pathway in PAH, we examined the protein expression of this signaling pathway in a rat model. The results showed that in the hypoxic PAH rat model, Brg1 expression was increased in the Hypoxia group, and Nrf2 was activated to undergo nuclear translocation, which increased the nuclear accumulation of Nrf2 and led to an increase in downstream HO-1 expression. We showed a decrease in the nuclear accumulation of Nrf2 and the expression of HO-1, but an increase in the cytoplasmic expression of Nrf2 in Brg1 knockdown rats compared with that of the Hypoxia group (Fig. 3B and D). In addition, the expression of anti-apoptotic protein Bcl-2 was increased and the expression of pro-apoptotic protein BAX was decreased in rats in the Hypoxia group compared with those in the Control group (Fig. 3E, F and G), suggesting that apoptosis was reduced and apoptosis resistance was generated, and apoptosis resistance was alleviated by loss of Brg1.

### Brg1 knockdown alleviated PAH progression in the lungs and right ventricle of MCT rats

**Fig. 4:**
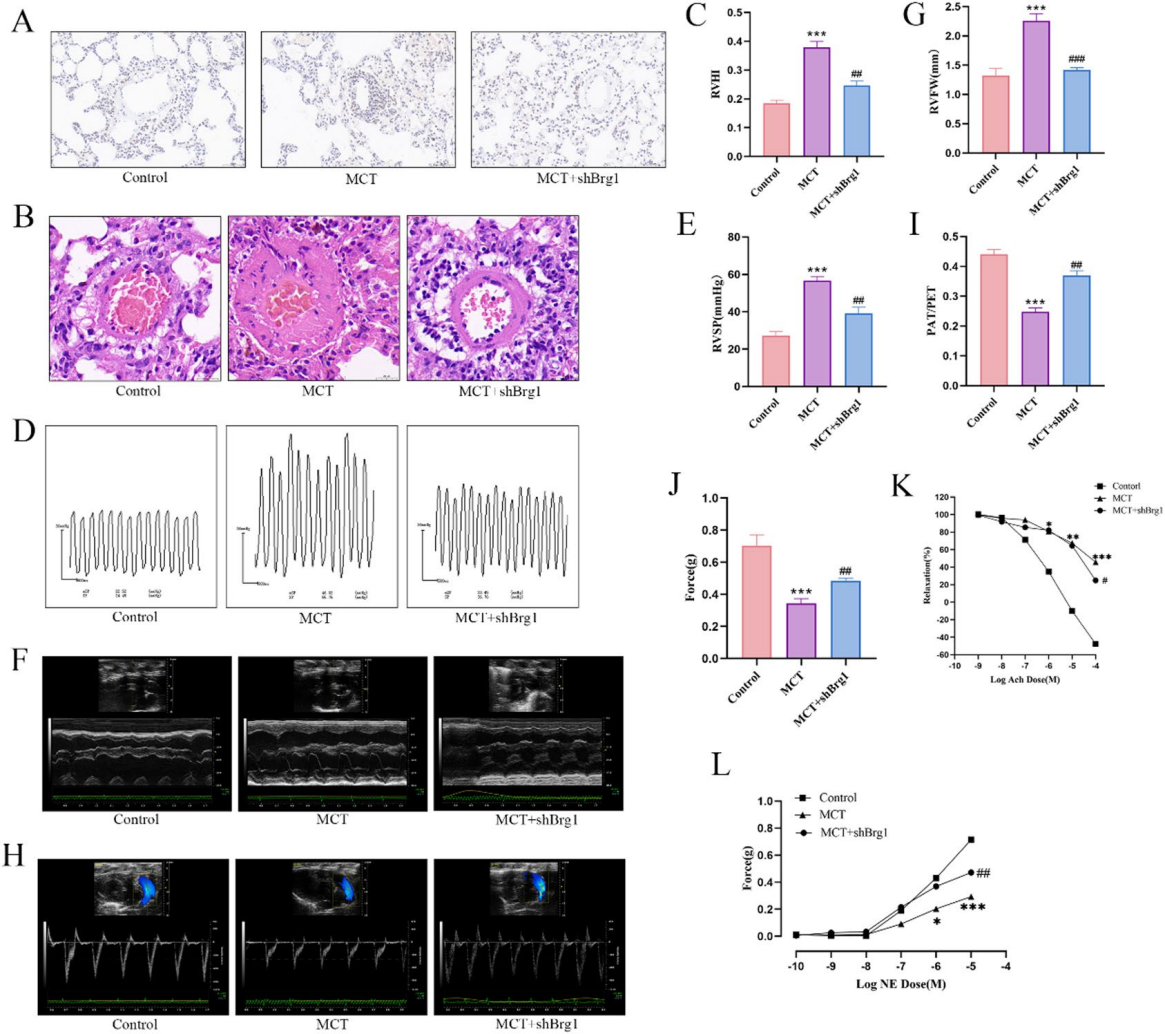
Brg1 knockdown alleviated PAH progression in the lungs and right ventricle of MCT rats. (**A**) Perivascular Brg1 expression in the lungs of rats in the Control, MCT, and MCT+shBrg1 groups. (**B**) HE staining results of rat lungs in each group. (**C**) Right ventricular hypertrophy index of rats in each group. (**D, E**) Graphs of RVSP and statistical analyses of rats in each group. (**F, G**) Graphs of right ventricular free wall thickness and statistical analyses of rats in each group. (**H, I**) Ratio of PAT to PET in each group of rats and graph of statistical analysis. (**J**) KCL-induced changes in pulmonary vasoconstriction. (**K**) Ach-induced changes in percentage of pulmonary vasodilation. (**L**) NE-induced changes in pulmonary vasoconstriction in each group of rats. Data was expressed as mean ± SD, *n* = 3. *Compared with Control group, *P < 0.05; **P < 0.01; ***P < 0.001; #Compared with MCT group, ^#^*P* < 0.05; ^##^*P* < 0.01; ^###^*P* < 0.001.

To explore the role of Brg1 in rat PAH, we established a rat model of PAH induced by MCT, and the same injection of adeno-associated virus knocked down the expression of Brg1. Immunohistochemistry of Brg1 in the lungs of control and MCT rats showed its expression in small pulmonary arterioles (Fig. 4A). Thickening of the wall of small pulmonary arteries was prevented in the MCT+shBrg1 group compared with the MCT group (Fig. 4B). In addition, injection of adeno-associated virus improved RV hypertrophy (Fig. 4C), RVSP (Fig. 4D and E), RVFW (Fig. 4F and G), and PAT/PET (Fig. 4H and I). The vascular ring results similarly showed that knockdown of Brg1 enhanced the contractility as well as the percentage of diastole of the pulmonary vasculature in MCT rats, significantly improving vascular tone in MCT rats (Fig. 4J, K and L). These results suggest that knockdown of Brg1 exert a therapeutic effect on cardiopulmonary function in MCT-induced PAH rats.

### Brg1 activated the Nrf2/HO-1 signaling pathway and promoted apoptosis resistance in PAH of MCT rats

**Fig. 5:**
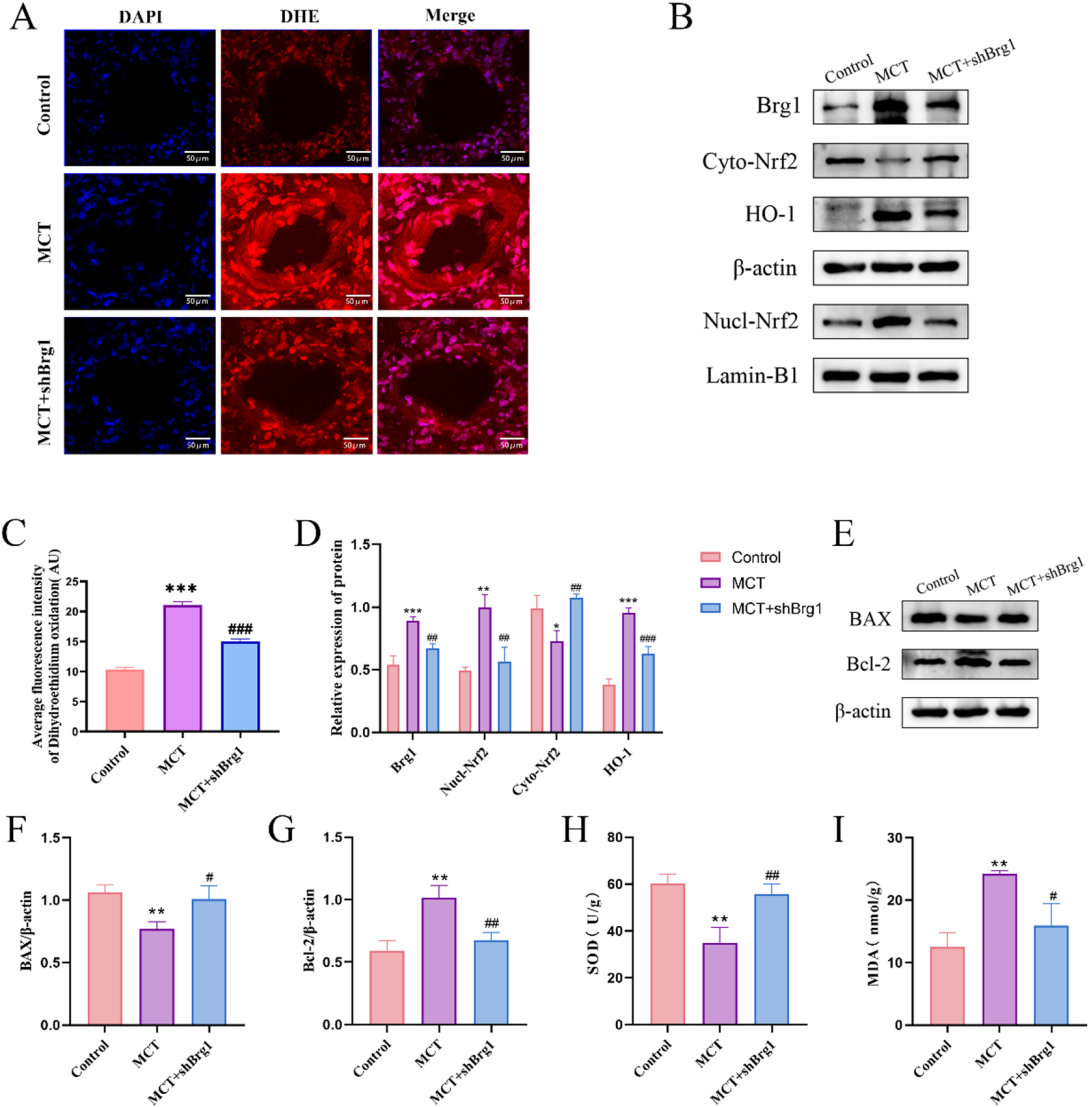
Brg1 activated the Nrf2/HO-1 signaling pathway and promoted apoptosis resistance in PAH of MCT rats. (**A**) Dihydroethidium (DHE) staining of pulmonary vessels was performed in the Control, MCT, and MCT+shBrg1 groups. (**B**) Western blot detection of Brg1, Nucl-Nrf2, Cyto-Nrf2, and HO-1 expression in lung tissues. (**C**) Statistical analysis of average fluorescence intensity of DHE. (**D**) Statistical analysis of Brg1, Nucl-Nrf2, Cyto-Nrf2, and HO-1 protein expression graph. (**E**) Western blot detection of Bcl-2, BAX expression in lung tissues. (**F**) Statistical analysis of BAX2 protein expression graph. (**G**) Statistical analysis of Bcl-2 protein expression graph. (**H**) SOD content in rat lung tissues. (**I**) MDA level in rat lung tissues. Data was expressed as mean ± SD, *n* = 3. *Compared with Control group, **P < 0.01; ***P < 0.001; ^#^Compared with MCT group, ^#^*P* < 0.05; ^##^*P* < 0.01; ^###^*P* < 0.001.

The level of small artery oxidative stress was evaluated in MCT rats. The results showed that the oxidative stress level of MCT group rats significantly increased, while knocking down Brg1 reduced the level of oxidative stress. (Figure 5A and C). We found that levels of MDA were significantly lower and SOD levels were significantly higher in the MCT+shBrg1 group than in the MCT group (Fig. 5H and I). Subsequently, we examined the expression of Nrf2/HO-1 pathway proteins in the MCT-induced PAH rat model, and Brg1 expression was increased in the MCT group, which led to nuclear accumulation of Nrf2 and increased HO-1 expression (Fig. 5B and D).

Next, we assessed changes in apoptosis in small pulmonary arteries of MCT PAH rats. Similarly, rats in the MCT group showed increased expression of the apoptosis-related protein Bcl-2 and decreased expression of BAX compared with rats in the Control group, suggesting that apoptosis was reduced and apoptosis resistance was generated, and the MCT+shBrg1 group showed decreased expression of Bcl-2 and increased expression of BAX compared with the MCT group (Fig. 5E, F and G), suggesting that knockdown of Brg1 attenuated apoptosis resistance.

### Brg1 promoted the proliferation of small pulmonary arteries in PAH rats

**Fig. 6:**
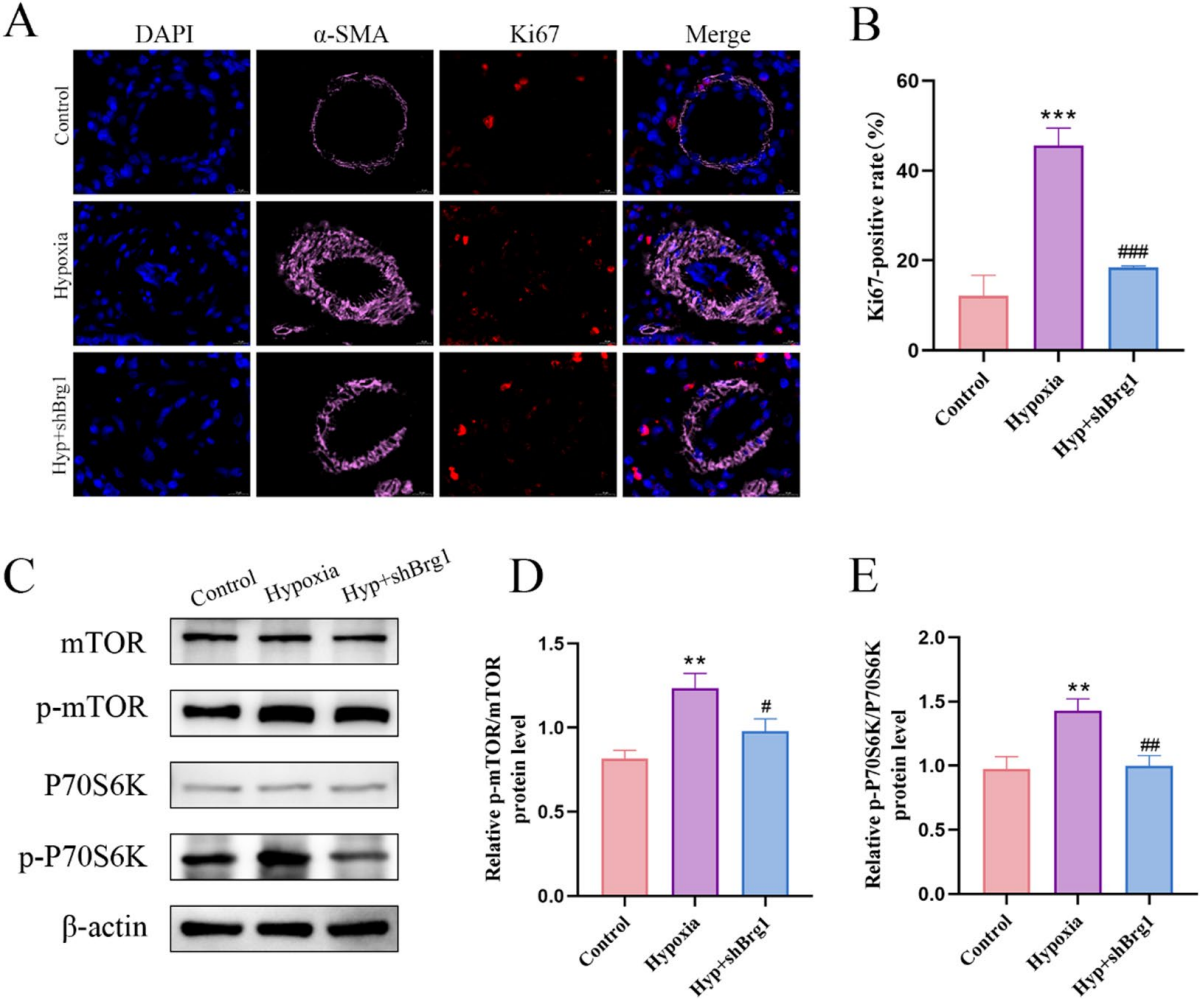
Brg1 promoted the proliferation of small pulmonary arteries in HPH rats. ( **A, B**) The expression of rat lung vascular smooth muscle proliferation in Control, Hypoxia, and Hyp+shBrg1 groups and statistical analysis graphs. (**C- E**) Western blot detection of mTOR, p-mTOR, P70S6K, p-P70S6K protein expression in rat lung tissues in Control, Hypoxia, and Hyp+shBrg1 groups and statistical analysis. Data was expressed as mean ± SD, *n* = 3. *Compared with Control group, **P < 0.01; ***P < 0.001; ^#^Compared with Hypoxia group, ^#^*P* < 0.05; ^##^*P* < 0.01; ^###^*P* < 0.001.

**Fig. 7:**
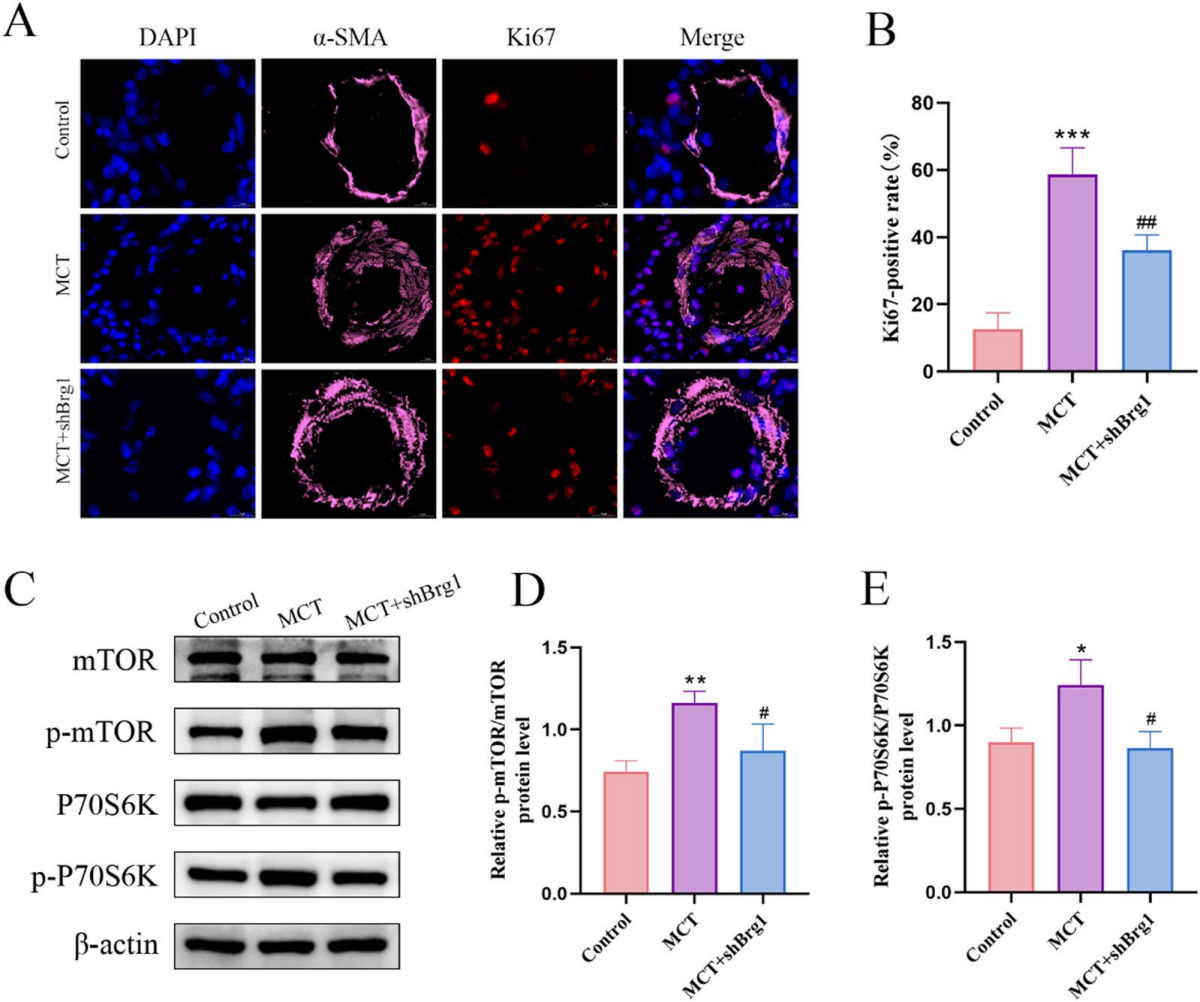
Brg1 promoted the proliferation of small pulmonary arteries in MCT-PAH rats. (**A**, **B**) Proliferation of rat lung vascular smooth muscle in Control, MCT, and MCT+shBrg1 groups and statistical analysis of the graph. (**C–E**) Western blot detection of mTOR, p-mTOR, P70S6K, p-P70S6K protein expression in rat lung tissues of Control group, MCT group, MCT+shBrg1 group and statistical analysis graph. Data was expressed as mean ± SD, *n* = 3. *Compared with Control group, *P < 0.05; **P < 0.01; ***P < 0.001; ^#^Compared with MCT group, ^#^*P* < 0.05; ^##^*P* < 0.01; ^###^*P* < 0.001.

To investigate the role of Brg1 in PAH proliferation, we examined the expression of Ki67 around small pulmonary arteries in lung tissue sections by immunofluorescence staining. The results showed that the expression of Ki67 in small pulmonary arteries was increased in lung tissue sections of rats in the Hypoxia group, indicating that the proliferation of small pulmonary arteries was increased in rats in the Hypoxia group, and Ki67 was significantly decreased after knocking down Brg1 (Fig. 6A and B). In the MCT-induced PAH model, Ki67 fluorescence results of lung tissue sections from the MCT+shBrg1 group showed reduced proliferation of small pulmonary arteries after knockdown of Brg1 compared with the MCT group (Fig. 7A and B). The Ki67 fluorescence results indicated that knockdown of Brg1 could reduce the proliferation of small pulmonary arteries.

The mTOR/P70S6K signaling pathway is mainly responsible for cell survival, proliferation, invasion, anti-apoptosis and promotion of angiogenesis^23^. To investigate the relationship between Brg1 and proliferation, we examined the expression of this pathway protein in rat lung tissues. The results showed that the protein levels of p-mTOR and p-P70S6K were significantly elevated in PAH rat compared with the control rats, whereas the total mTOR and P70S6K proteins were barely changed. The p-mTOR and p-P70S6K expression was obviously suppressed after knockdown of Brg1, while the total protein expression of mTOR and P70S6K were barely changed (Fig. 6C, D and E). In the MCT-induced PAH model, protein levels of both p-mTOR/mTOR and p-P70S6K/ P70S6K were increased in the MCT group compared with the Control group, and the p-mTOR/mTOR and p-P70S6K/P70S6K ratios were decreased after knockdown of Brg1 (Fig. 7C, D and E). These results suggest that Brg1 is involved in proliferation by regulating the mTOR /P70S6K signaling pathway.

### Brg1 promoted Nrf2 nuclear translocation in PASMCs, and Brg1 knockdown attenuated oxidative stress levels in PAH-PASMCs

**Fig. 8:**
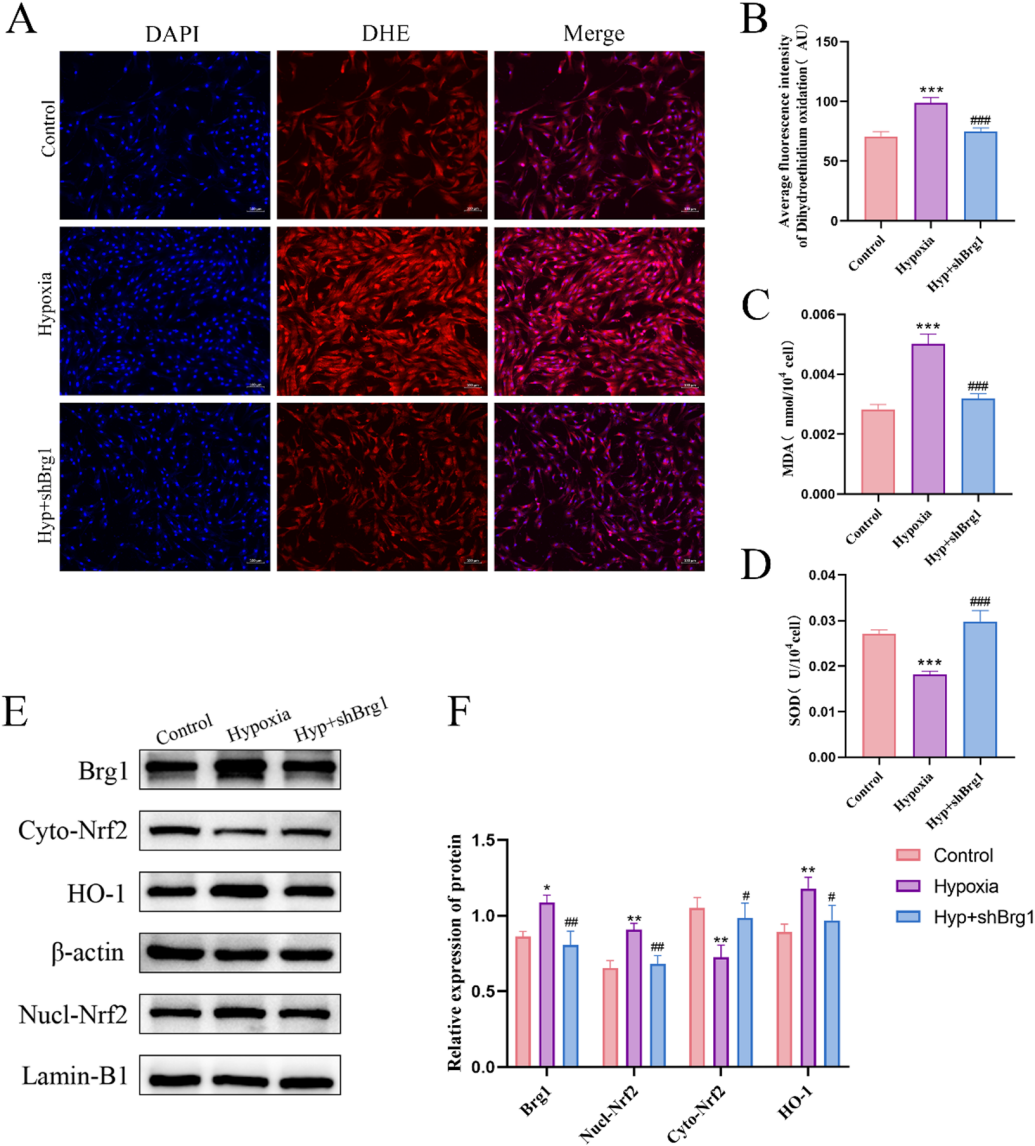
Brg1 promoted Nrf2 nuclear translocation in PASMCs, and Brg1 knockdown attenuates oxidative stress levels in PAH-PASMCs. (**A**, **B**) DHE staining detection of oxidative stress level in PASMCs and statistical analysis graph. (**C**) MDA level of PASMCs in each group. (**D**) SOD content of PASMCs in each group. (**E, F**) Western blot detection of Brg1, Nucl-Nrf2, Cyto-Nrf2, and HO-1 expression in PASMCs and statistical analysis graph. Data was expressed as mean ± SD, *n* = 3. *Compared with Control group, *P < 0.05; **P < 0.01; ***P < 0.001; ^#^Compared with Hypoxia group, ^#^*P* < 0.05; ^##^*P* < 0.01; ^###^*P* < 0.001.

To further investigate the role of Brg1 in PAH, we isolated PASMCs from the pulmonary arteries of 6-8-week-old SD rats and cultured under hypoxic conditions to mimic PAH in vitro. DHE fluorescence staining showed that the level of oxidative stress was elevated in the Hypoxia group and decreased in the Hyp+shBrg1 group (Fig. 8A and B). Next, we examined the SOD content and MDA level, indicators related to oxidative stress, in PASMCs, and the results showed that compared with the Hypoxia group, the Hyp+shBrg1 group had a decreased MDA level and increased SOD content, indicating a decreased level of oxidative stress (Fig. 8C and D), which was consistent with the results of the in vivo experiments.

We showed that Brg1 expression was significantly elevated under hypoxic conditions, and adenoviral transfection effectively knocked down Brg1 expression. Meanwhile, we found Brg1 promoted nuclear translocation of Nrf2 and increased Nrf2 of the nucleus and HO-1 protein expression, while decreased cytoplasmic Nrf2 expression, which abrogated by loss of Brg1 (Fig. 8E and F).

### Loss of Brg1 reduced the proliferation, migration capacity and anti-apoptotic phenotype of PAH-PASMCs

**Fig. 9:**
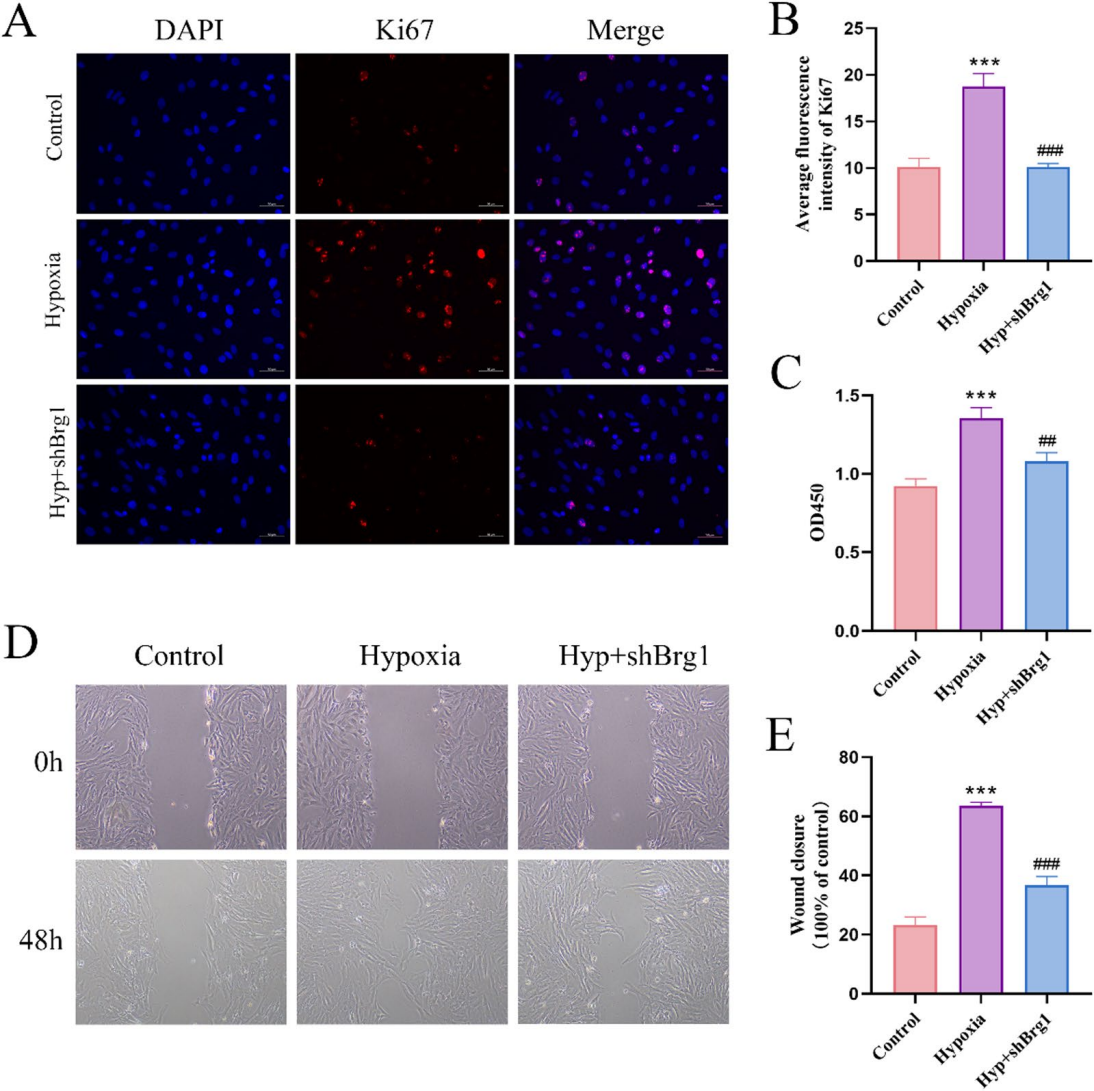
Loss of Brg1 reduced the proliferation and migration capacity of PAH-PASMCs. (**A**, **B**) Ki67 immunofluorescence typical images and statistical analysis of each group. (**C**) CCK-8 assay to detect cell proliferation. (**D, E**) Wound healing assay typical images and statistical analysis of each group. Data was expressed as mean ± SD, *n* = 3. *Compared with Control group, **P < 0.01; ***P < 0.001; ^#^Compared with Hypoxia group, ^#^*P* < 0.05; ^##^*P* < 0.01; ^###^*P* < 0.001.

**Fig. 10:**
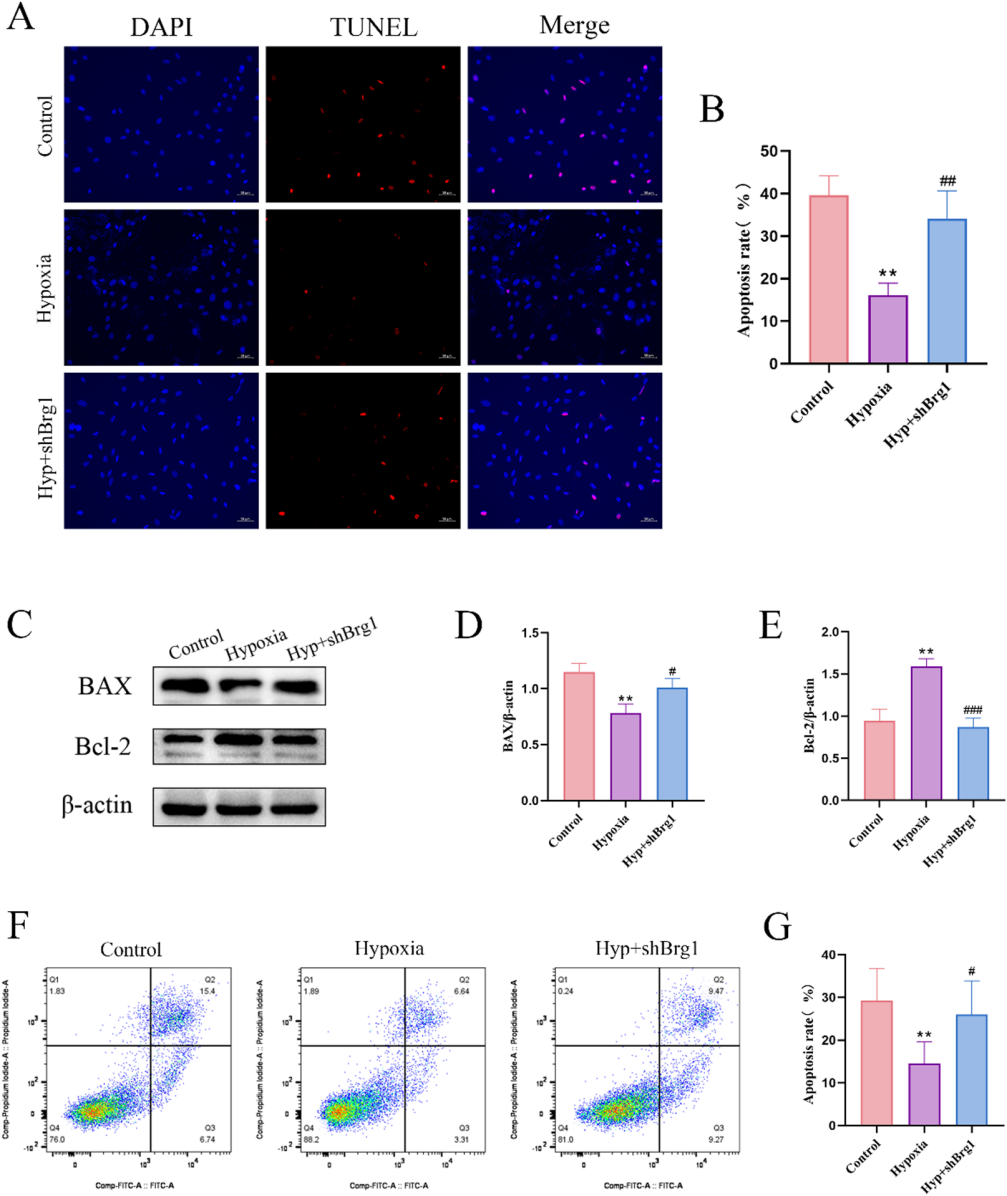
Loss of Brg1 reduced the anti-apoptotic phenotype of PAH-PASMCs. (**A**, **B**) TUNEL fluorescence typical images and statistical analysis of each group. (**C–E**) Western blot for the detection of Bcl-2 and BAX expression in PASMCs and statistical analysis of each group. (**F, G**) Flow cytometry detection of apoptosis in each group and statistical analysis of each group. Data was expressed as mean ± SD, *n* = 3. *Compared with Control group, **P < 0.01; ***P < 0.001; ^#^Compared with Hypoxia group, ^#^*P* < 0.05; ^##^*P* < 0.01; ^###^*P* < 0.001.

Ki67 fluorescence results showed decreased proliferation ability in the Hyp+shBrg1 group compared to the Hypoxia group (Fig. 9A and B). Consistently, CCK-8 experiments showed the same results (Fig. 9C). In addition, we performed a wound healing assay to monitor the wound healing ability between the different groups. The results showed enhanced wound healing ability in the Hypoxia group and greatly reduced wound healing ability in the Hyp+shBrg1 group (Fig. 9D and E). Thus, these results suggest that PASMCs exhibit enhanced migration and proliferation abilities through increased Brg1 expression, and decreased migration and proliferation abilities after knockdown of Brg1. Moreover, the apoptosis was significantly decreased in the Hypoxia group, while increased in the Hyp+shBrg1 group compared with the Control group (Fig. 10A and B). Similarly, Western blot results showed that the expression of apoptosis-related protein Bcl-2 was decreased and BAX was increased in the Hyp+shBrg1 group compared with the Hypoxia group (Fig. 10C, D and E), suggesting that its apoptosis resistance was suppressed. Furthermore, flow cytometry showed a significant increase in the number of apoptotic cells after knockdown of Brg1.(Figure 10F and G).

### Brg1 mediated Nrf2 regulation of cell proliferation via the mTOR /P70S6K pathway

**Fig. 11:**
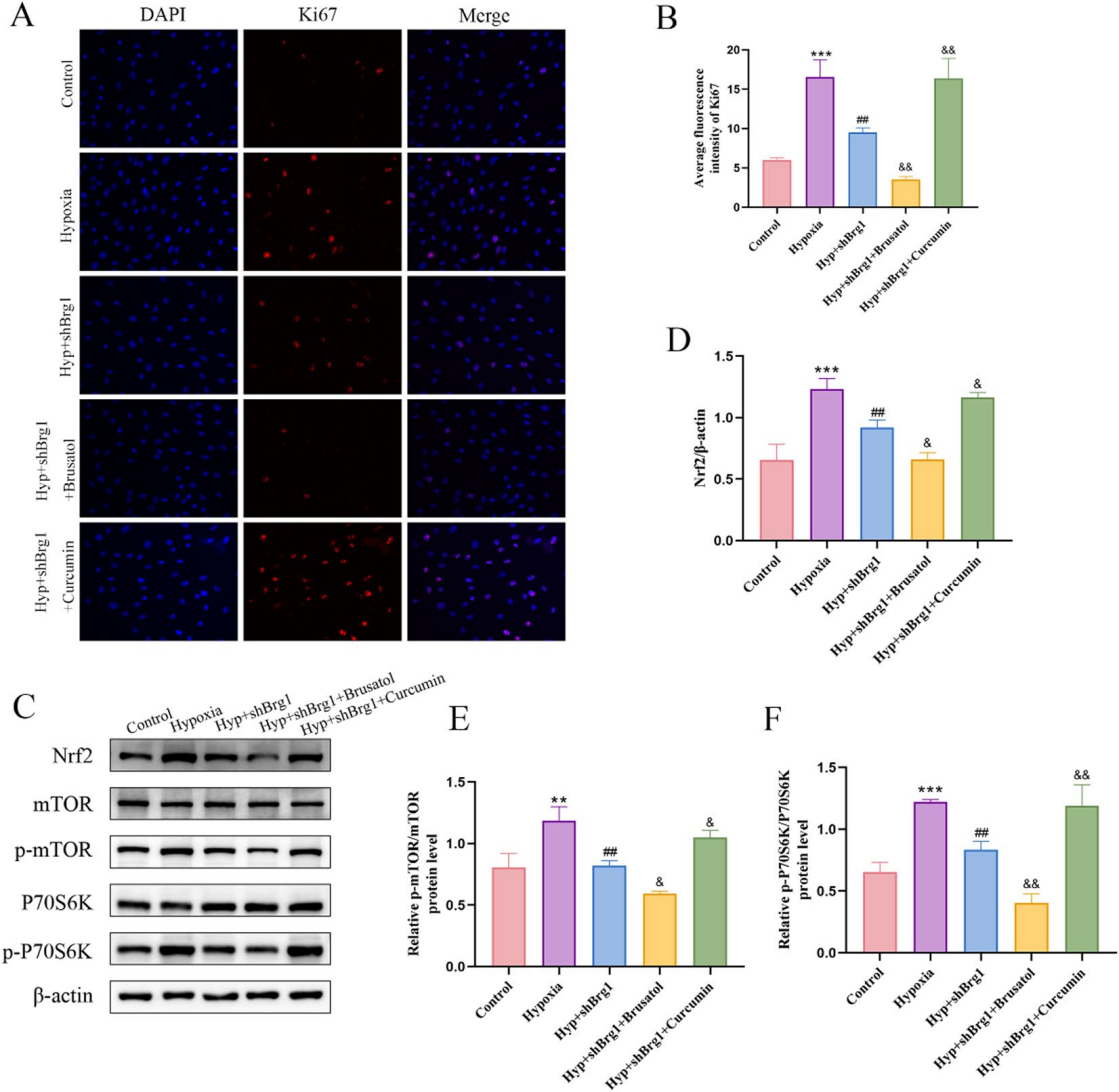
Brg1 mediated Nrf2 regulation of cell proliferation via the mTOR /P70S6K pathway. (**A, B**) Typical images of Ki67 immunofluorescence and statistical analyses for each group. (**C–F**) Western blot detection of Nrf2, mTOR, p-mTOR, P70S6K, p-P70S6K protein expression in PASMCs and statistical analysis. Data was expressed as mean ± SD, *n* = 3. *Compared with Control group, ***P < 0.001; ^#^Compared with Hypoxia group, ^##^*P* < 0.01; ^&^Compared with Hyp+shBrg1 group, ^&^*P* < 0.05; ^&&^*P* < 0.01.

We performed Ki67 immunofluorescence assay to detect the proliferation of smooth muscle cells in each group after addition of Nrf2 inhibitor Brusatol and Nrf2 agonist Curcumin (Fig. 11A and B). The results showed that cell proliferation was further inhibited in the Brusatol addition compared to the Hyp+shBrg1 group, and increased with the addition of Curcumin.

To further explore the relationship between Nrf2 and the proliferation of PASMCs, we investigated the effect of Nrf2 on the mTOR /P70S6K proliferation signaling pathway (Fig. 11C). The results showed that Nrf2 protein expression was increased under hypoxic conditions (Fig. 11D), while the protein level of p-mTOR was significantly elevated compared to the total mTOR level (Fig. 11E), and the protein level of p-P70S6K was also significantly elevated compared to the total P70S6K level (Fig. 11F). Nrf2, as well as p-mTOR, and p-P70S6K expression was inhibited after knockdown of Brg1, and the total protein expression was unchanged. Nrf2 as well as p-mTOR, p-P70S6K protein expression was further suppressed by the addition of Brusatol, while the addition of Curcumin alleviated the decrease in Nrf2 expression caused by the knockdown of Brg1, while p-mTOR, p-P70S6K protein expression was also elevated as a result. The above experimental results suggest that Brg1 can regulate the PASMCs mTOR /P70S6K pathway and affect the cell proliferation phenotype by mediating Nrf2.

## Discussion

**Fig. 12:**
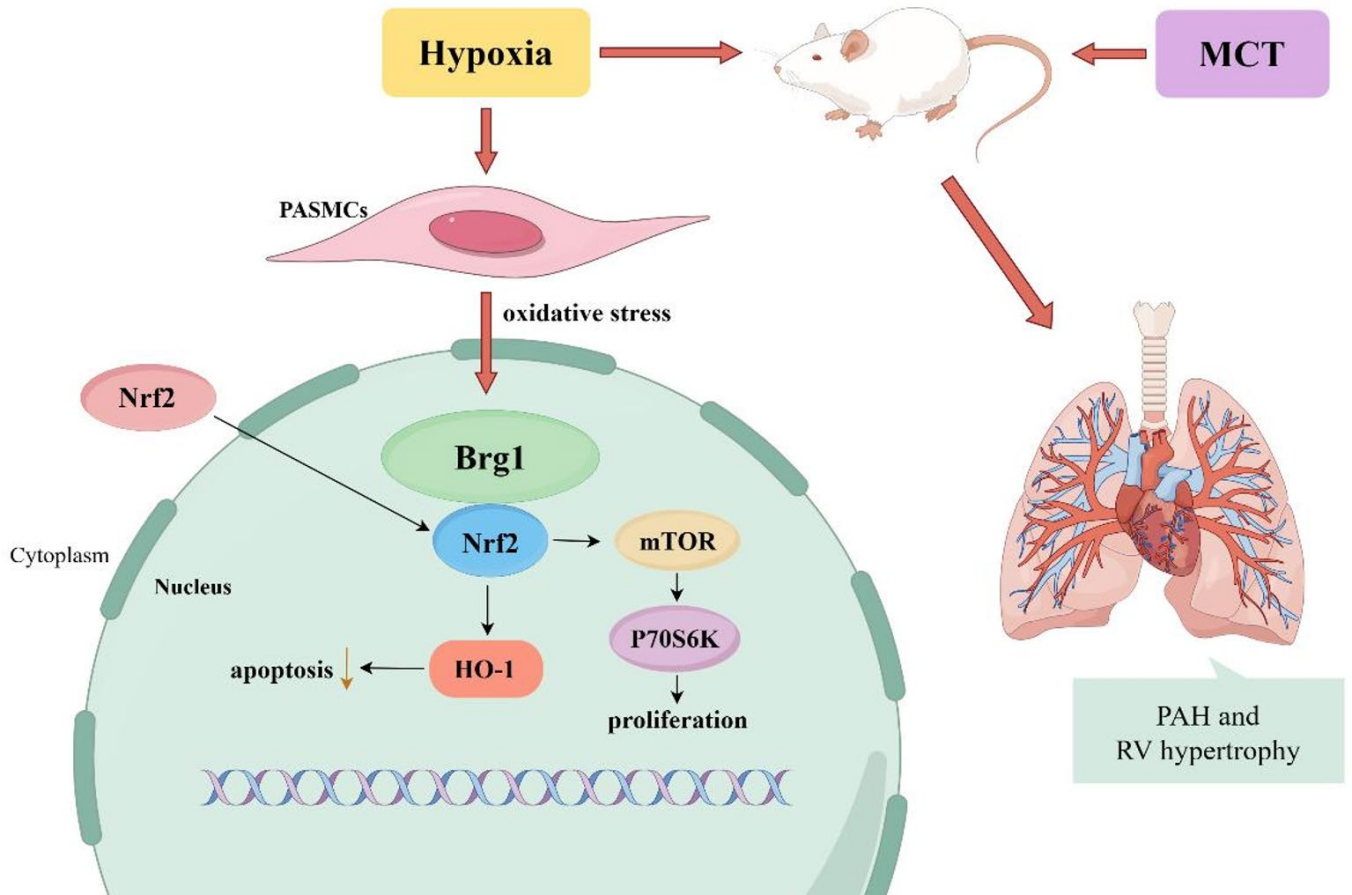
Mechanism diagram. Normal SD rats stimulated by hypoxia or given the intervention of injected MCT developed smooth muscle proliferation in small pulmonary arteries, leading to pulmonary hypertension, which in turn led to right ventricular hypertrophy and right heart failure. Similarly, oxidative stress occurs when PASMCs are stimulated by hypoxia, leading to increased expression of the chromatin remodeling factor Brg1, which allows nuclear translocation of Nrf2 and activation of the Nrf2/HO-1 signaling pathway to increase apoptosis resistance. Meanwhile, Brg1 and Nfr2 can activate their downstream proliferation-associated signaling pathway mTOR/P70S6K to cause proliferation of PASMCs, leading to the progression of pulmonary hypertension.

In the present study, we revealed the important role of Brg1 in PAH. The results showed that up-regulation of Brg1 expression in lung tissues was confirmed in a model of hypoxic pulmonary hypertension and a model of MCT-induced pulmonary hypertension, and the same results were obtained in in vitro experiments with extracted primary rat PASMCs subjected to hypoxia. PAH rats exhibited elevated right ventricular hypertrophy index, increased right ventricular and cross-sectional area, impaired cardiac function, and elevated pulmonary artery pressure, which were significantly improved by knockdown of Brg1. In addition, the level of oxidative stress was increased in the PAH model group, and the Nrf2/HO-1 signaling pathway was activated and accompanied by an increase in the proliferation of the vascular smooth muscle layer, which produced apoptosis resistance. Knockdown of Brg1 attenuates oxidative stress in PAH in vivo and in vitro and attenuates proliferation and apoptosis resistance in PASMCs. It suggests that knockdown of Brg1 has a mitigating effect on PAH, which may be a potential target for the treatment of PAH. The proposed mechanism is illustrated in the figure (Fig. 12).

The SWI/SNF complex is a highly conserved multisubunit complex in eukaryotes that is involved in chromatin remodeling for dynamic regulation of gene expression and plays a crucial role in gene regulation, differentiation, DNA repair and development^24^. Brm (Brahma/SMARCA2) and Brg1 are two mutually exclusive ATPases in the SWI/SNF complex capable of inducing chromatin changes to regulate gene transcription^25^. It has been shown that Brg1 promotes the expression of hypoxia-inducible factor (HIF), which facilitates the induction of HIF1 and its hypoxia pathway, thereby regulating the hypoxic response^26^. Hypoxia-inducible factor is a regulator of various cellular stress processes^27,28^, so we can speculate that Brg1 also plays a key role in regulating PAH. Regarding the role of Brg1 in oxidative stress, it has been shown in studies related to other diseases that Brg1 promotes ROS production and endothelial mesenchymal transition, leading to hepatic fibrosis in mice^29^, that Brg1 deficiency attenuates NAFLD in mice by attenuating ROS production in hepatocytes^30^, and that Brg1 regulates trans-activation of NOX in endothelial cells and leads to myocardial ischemia-reperfusion injury^31^. In our current study, we found that hypoxic conditions upregulated Brg1 protein expression in PASMCs in vivo and in vitro, leading to increased levels of oxidative stress, and reduced levels of oxidative stress after knockdown of Brg1, suggesting that Brg1 may be one of the key factors affecting oxidative stress in PAH.

Nrf2 acts as a central transcription factor of the endogenous antioxidant pathway and regulates the expression of various antioxidant enzymes in vivo^32^. It has been shown that Nrf2 overexpression effectively increases antioxidant levels, promotes cell proliferation, and is able to inhibit the intrinsic apoptotic pathway in PDLSCs by suppressing active caspase-9 and BAX^33^. Heme oxygenase-1 (HO-1) is an oxygen-dependent enzyme that regulates vascular tone and cell proliferation and is promoted by hypoxia^34^. HO-1 regulates a variety of metabolic processes such as apoptosis, anti-inflammation, and oxidative stress^35^. Brg1 promotes Nrf2 recruitment and up-regulates HO-1 expression^36^. Brg1-mediated Nrf2/HO-1 signaling has been reported to be involved in a variety of pathological processes. In the present study, elevated Brg1 expression in the Hypoxia and MCT groups resulted in the activation of Nrf2 for nuclear translocation in the PAH model. However, it is worth noting that since the production of HO-1 is not sufficient to attenuate oxidative stress as well as to scavenge excess reactive oxygen species^37^, the level of oxidative stress remains elevated in the rat model Hypoxia group and the MCT group due to the inability to scavenge excess ROS, despite the increased expression of HO-1 protein. The results in the in vitro PASMCs assay were consistent with the in vivo results.

Apoptosis is a programmed cell death process that occurs when intracellular death cascades are triggered by specific signals, and plays an important role in a variety of diseases. It has been suggested that LSPCs can activate Nrf2/HO-1 to inhibit the mitochondria-mediated apoptotic signaling pathway thereby enhancing cellular antioxidant defense^38^. Studies in past cardiomyocytes have shown that overexpression of Brg1 activates the Nrf2/HO-1 signaling pathway contributing to the inhibition of cardiomyocyte apoptosis and the promotion of damaged cardiomyocyte survival in infarction models^39^. Nrf2 deficiency also leads to an increased rate of EC apoptosis and induces an impairment of structural integrity of newly formed capillaries^40^, which has emerged as a novel mechanism of vascular aging. In our experimental results, we found that the elevated expression of Brg1 could indeed promote the expression of anti-apoptotic protein Bcl-2, and at the same time inhibit the expression of pro-apoptotic protein BAX, which was manifested in the thickening of pulmonary vascular smooth muscle layer due to apoptosis resistance in small pulmonary arteries as well as in PASMCs, and the TUNEL results also showed that Brg1 could inhibit apoptosis in PASMCs. These data suggest that Brg1 activates the Nrf2/HO-1 signaling pathway and contributes to the antiapoptotic capacity of small pulmonary arteries, leading to vascular remodeling and exacerbation of PAH.

A major cause of PAH is pulmonary vascular remodeling due to hyperproliferation and apoptosis resistance of pulmonary arterial smooth muscle^41^. Under the influence of hypoxia, inflammation, and shear stress of blood flow, cells have increased ROS production and decreased scavenging capacity, elevated levels of oxidative stress thereby causing an imbalance in calcium-ion homeostasis in vascular smooth muscle, and increased production of vascular growth factor, which leads to proliferation of vascular smooth muscle^42,43^. The SWI/SNF complex is a key epigenetic effector in the orderly regulation of cell proliferation and differentiation^44^. Previously, the effect of Brg1 on cell proliferation has been studied more in cancer, and in prostate cancer^45^, hepatocellular carcinoma^13^, lung cancer^46^, and pancreatic cancer^26^, the overexpression of Brg1 enhanced the proliferation and migration of tumor cells and invasion, and the knockdown of Brg1 was able to inhibit the proliferation and migration of cancer cells. Meanwhile, pulmonary vascular cells in PAH all showed tumor-like properties, such as excessive proliferation and migration, hypersecretion, and metabolic abnormalities in both endothelial cells and smooth muscle cells^47^. The role of Brg1 on cell proliferation in PAH is unknown. In our cellular experimental results, PASMCs exhibited high proliferation and migration capacity under hypoxic conditions, and Brg1 knockdown effectively reduced the proliferation and migration capacity of PAH-PASMCs, and these results are consistent with previous findings of Brg1 in other diseases.

mTOR regulates a variety of cellular processes such as cell growth, migration and differentiation^48^. P70S6K is an important target protein related to metabolism downstream of mTOR and is involved in a variety of cellular activities. Briefly, activation of the mTOR/P70S6K signaling pathway promotes smooth muscle cell dedifferentiation and proliferation^49^. Drug targeting of the mTOR /P70S6K signaling pathway can inhibit vascular growth factor-induced smooth muscle cell proliferation, dedifferentiation and migration^50^. In our experiments, to investigate how Brg1 is involved in the proliferative phenotype of PASMCs, we first examined the protein expression of the mTOR /P70S6K signaling pathway in rat lung tissues, and the results showed that knockdown of Brg1 inhibited proliferation. Some scholars^51^ suggested that dysregulated Nrf2 may promote cell proliferation and trigger angiogenesis. Next, in order to further explore the involvement of Brg1-mediated Nrf2 in cell proliferation, we silenced Brg1 by adding the Nrf2 inhibitor Brusatol and the Nrf2 agonist Curcumin and then detected the changes in the expression of proteins related to the mTOR/P70S6K pathway. The results showed that the addition of Brusatol further enhanced the inhibitory effect of silencing Brg1 on the proliferation of PASMCs.

Unfortunately, there are some limitations of our experiments. The current study mainly used the Brg1 knockdown strategy, and the lack of function acquisition or rescue experiments is a limiting factor that will be further refined in the future. In hemodynamic experiments we focused more on RVSP and RVHI as markers of disease severity. In the development of PAH, right ventricular systolic pressure (RVSP) is highly correlated with pulmonary artery systolic pressure (PASP). We measured only RVSP and missing data from experiments on mean pulmonary arterial pressure (mPAP) and transcatheter pulmonary valve replacement (TPVR) in rats. We will continue to improve the technique to synchronize the measurement of pulmonary artery pressure (PAP) and RVSP in the future. In addition, we used only male rats in the present study mainly to exclude the possible confounding factors of sex hormone fluctuations, such as estrogen, on the development of pulmonary arterial hypertension, and thus to reduce the variability of the experimental results. We now recognize this limitation and will include female animals in future studies to comprehensively assess the mechanisms of the Brg1-Nrf2 signaling axis in the context of different sexes, thus improving the generalizability and scientific validity of our findings.

In summary, our study reveals that knockdown of Brg1 alleviates RV dysfunction and reduces pulmonary vascular remodeling in PAH. Brg1 is able to activate the Nrf2/ HO-1 oxidative damage-related signaling pathway to exert anti-apoptotic effects. In addition, changes in the mTOR /P70S6K pathway indicated that Brg1 could mediate Nrf2 to regulate the proliferation of PASMCs to promote the progression of PAH. These findings may provide new insights for future therapeutic strategies to control PAH, but the specific mechanisms must be further explored.

## Supplementary Information

Below is the link to the electronic supplementary material.

Supplementary Material 1

Supplementary Material 2

## Data Availability

Te datasets generated and analyzed during the current study are available from the corresponding author on reasonable request.

## Abbreviations

Brg1: Brahma-related gene 1
PAH: Pulmonary arterial hypertension
MCT: Monocrotaline
Nrf2: Nuclear factor erythroid-2 related factor 2
PASMCs: Pulmonary artery smooth muscle cells
Hyp: Hypoxia
ARE: Antioxidant response element
MDA: Malondialdehyde
SD: Sprague-Dawley
SOD: Superoxide dismutase
HIF: Hypoxia-inducible factor
RVFW: Right ventricle free wall
PAT: Pulmonary acceleration time
PET: Pulmonary ejection time
RVSP: Right ventricular systolic pressure
RV: Right ventricular
HE: Hematoxylin-eosin
RVHI: Right ventricular hypertrophy index
BSA: Bovine serum albumin
RHC: Right heart catheter
DHE: Dihydroethidium
