# Supplementary material for: Loss of Brg1 prevents the progression of pulmonary hypertension by inhibiting Nrf2 expression
Source: Sci Rep. 2026 Jun 3;16:25395. doi: 10.1038/s41598-026-55301-6 (PMC13473476; doi:10.1038/s41598-026-55301-6)

Figure1

C

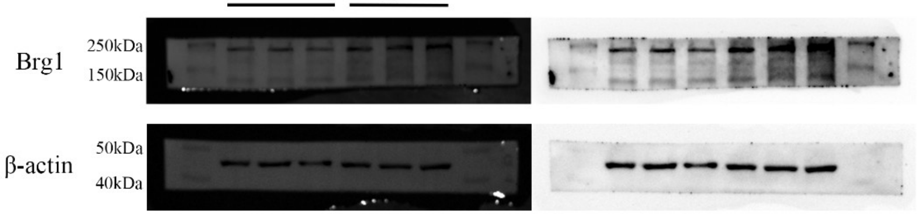

E

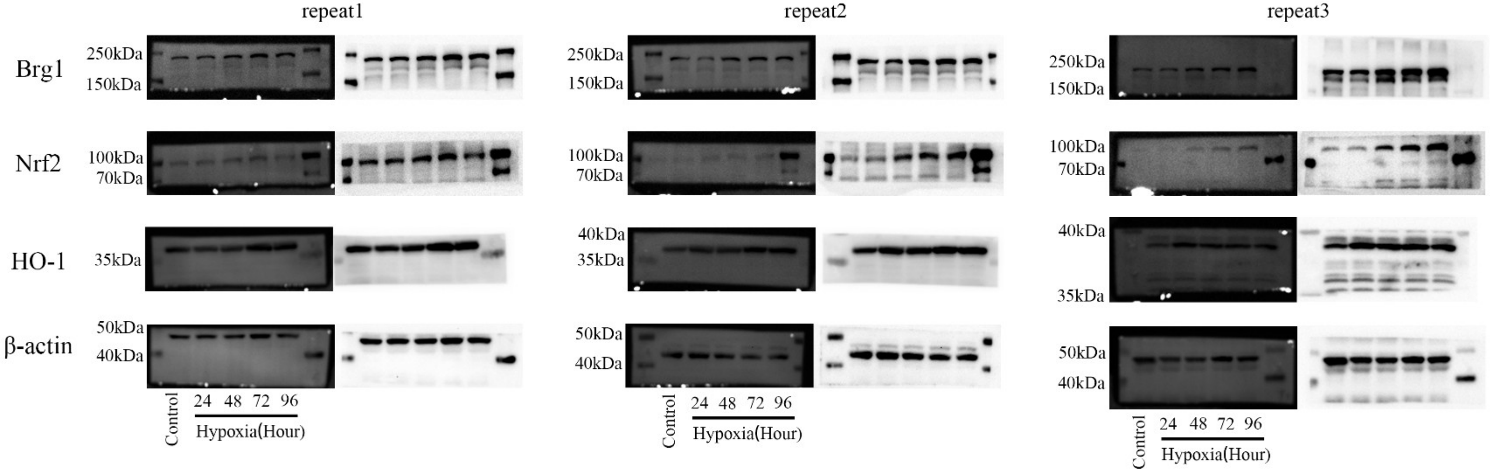

Full blots

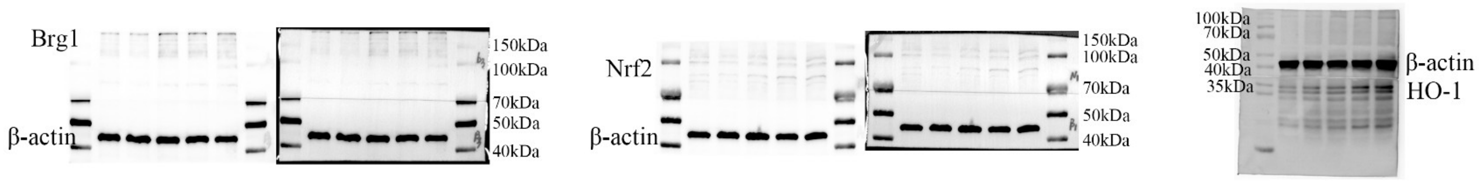

Figure3

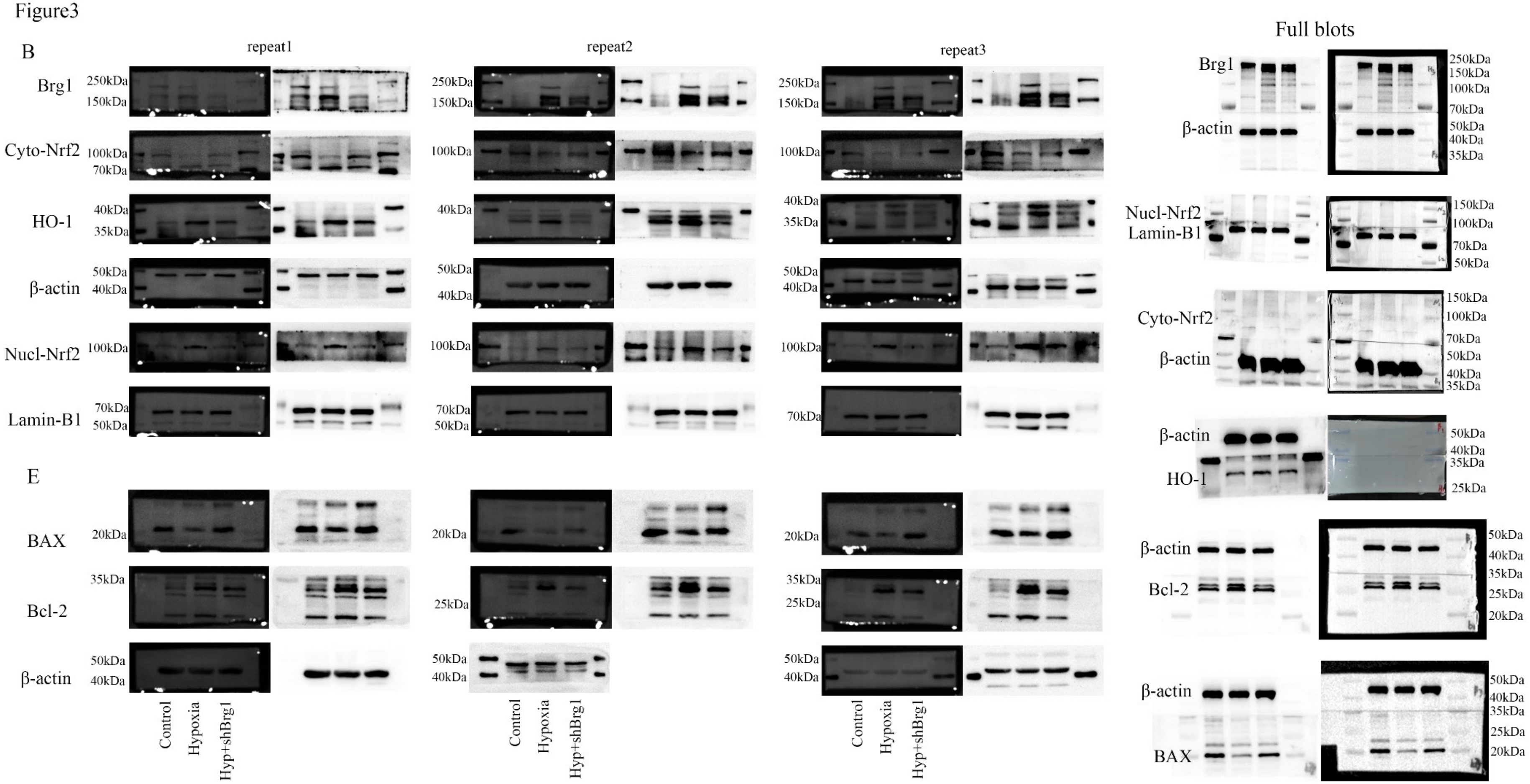
B
repeat1
repeat2
repeat3
Full blots
Brg1
Cyto-Nrf2
HO-1
β-actin
Nucl-Nrf2
Lamin-B1
E
BAX
Bcl-2
β-actin
250kDa
150kDa
100kDa
70kDa
50kDa
40kDa
35kDa
25kDa
20kDa
Control
Hypoxia
Hyp+shBrg1

Figure5

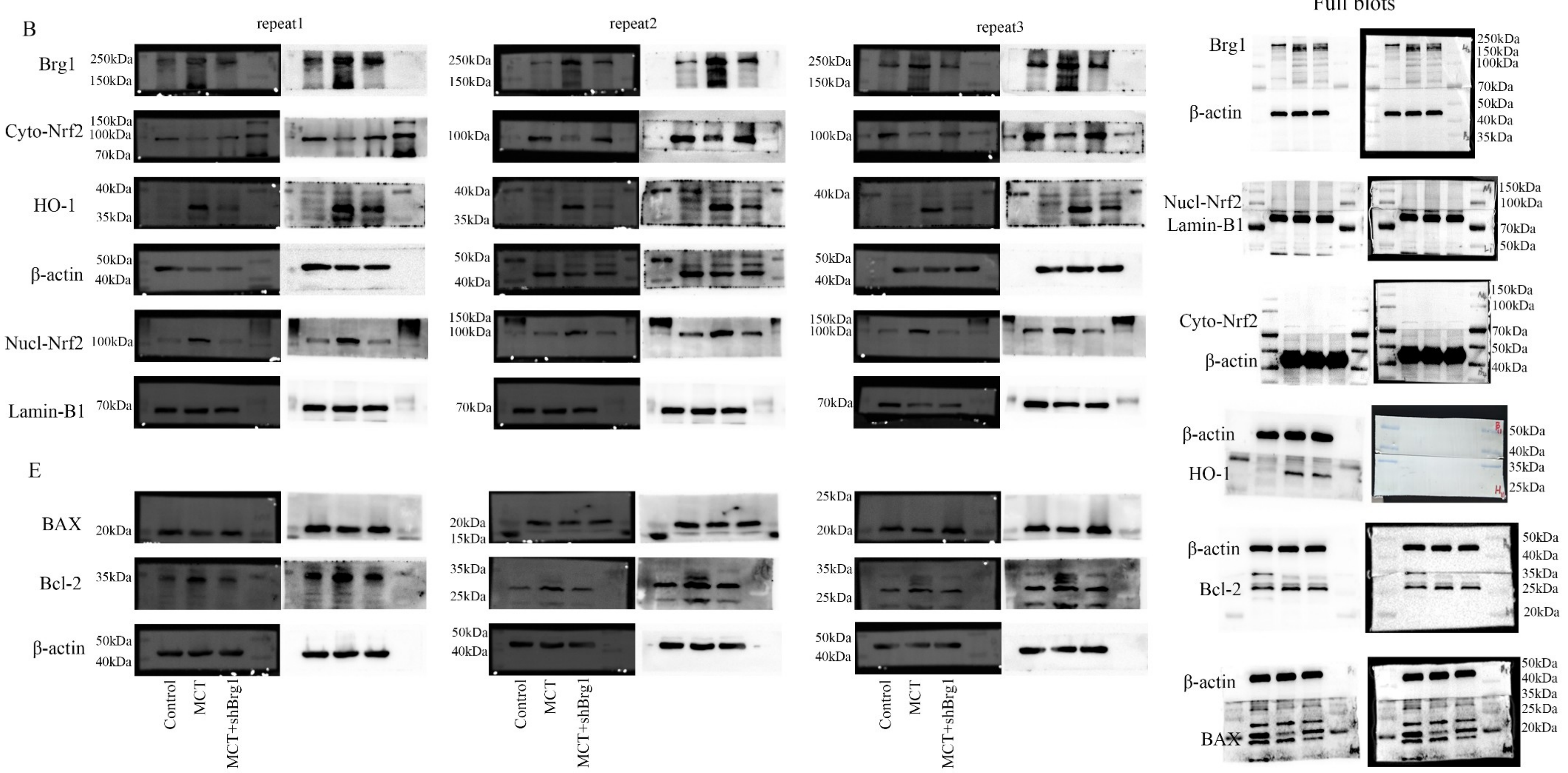
B
repeat1
repeat2
repeat3
Full blots
Brg1
Cyto-Nrf2
HO-1
β-actin
Nucl-Nrf2
Lamin-B1
E
BAX
Bcl-2
β-actin
Control
MCT
MCT+shBrg1
250kDa
150kDa
100kDa
70kDa
50kDa
40kDa
35kDa
25kDa
20kDa
15kDa

Figure6

C

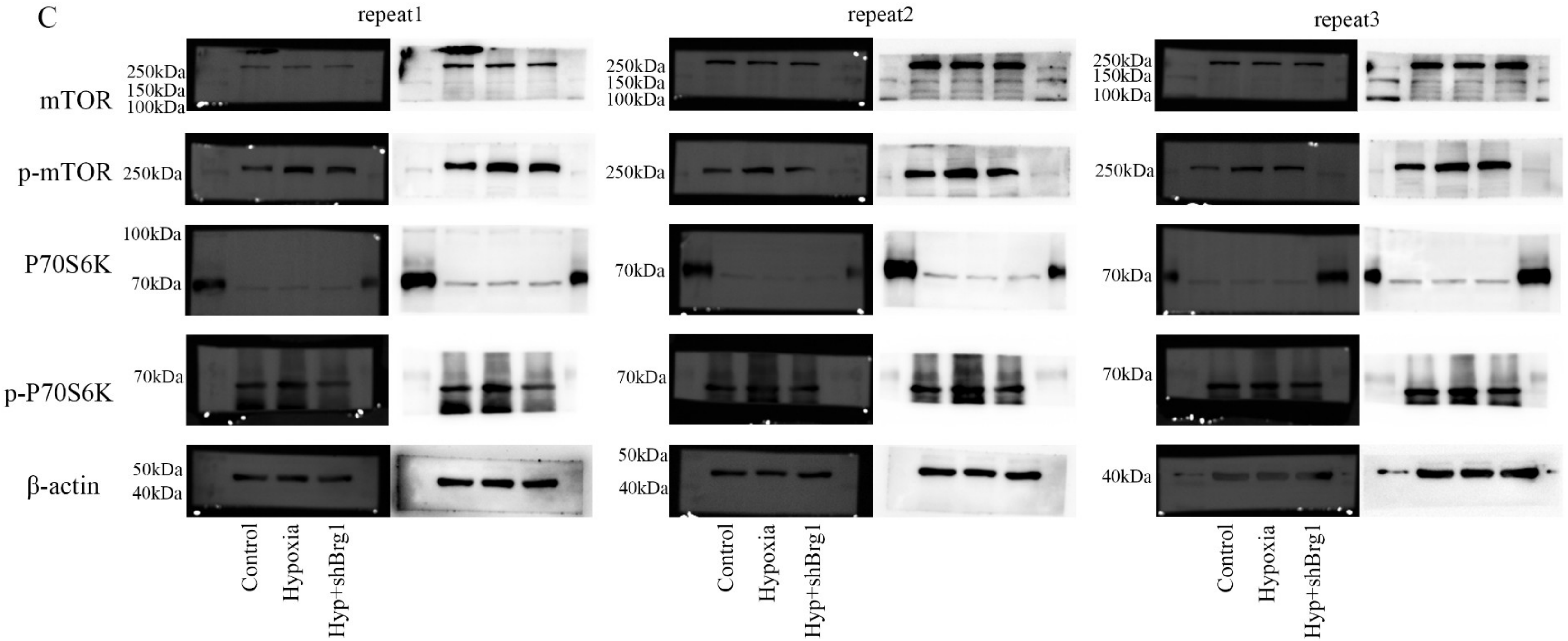

Full blots

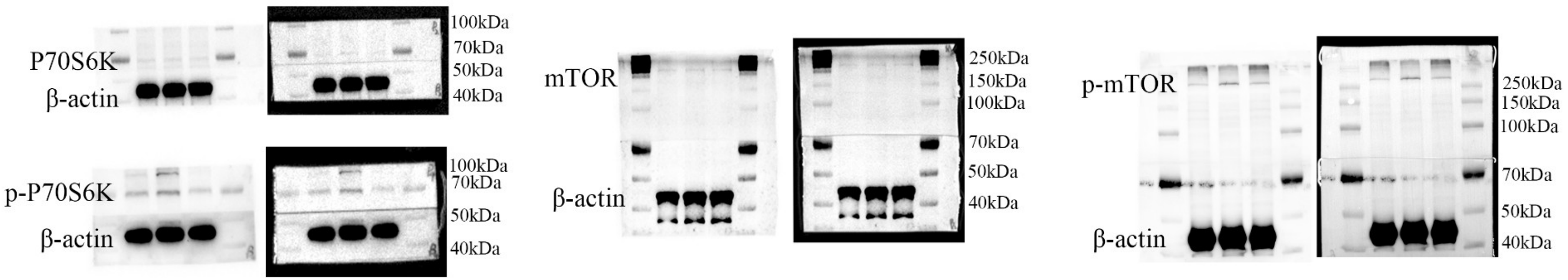

Figure8

E

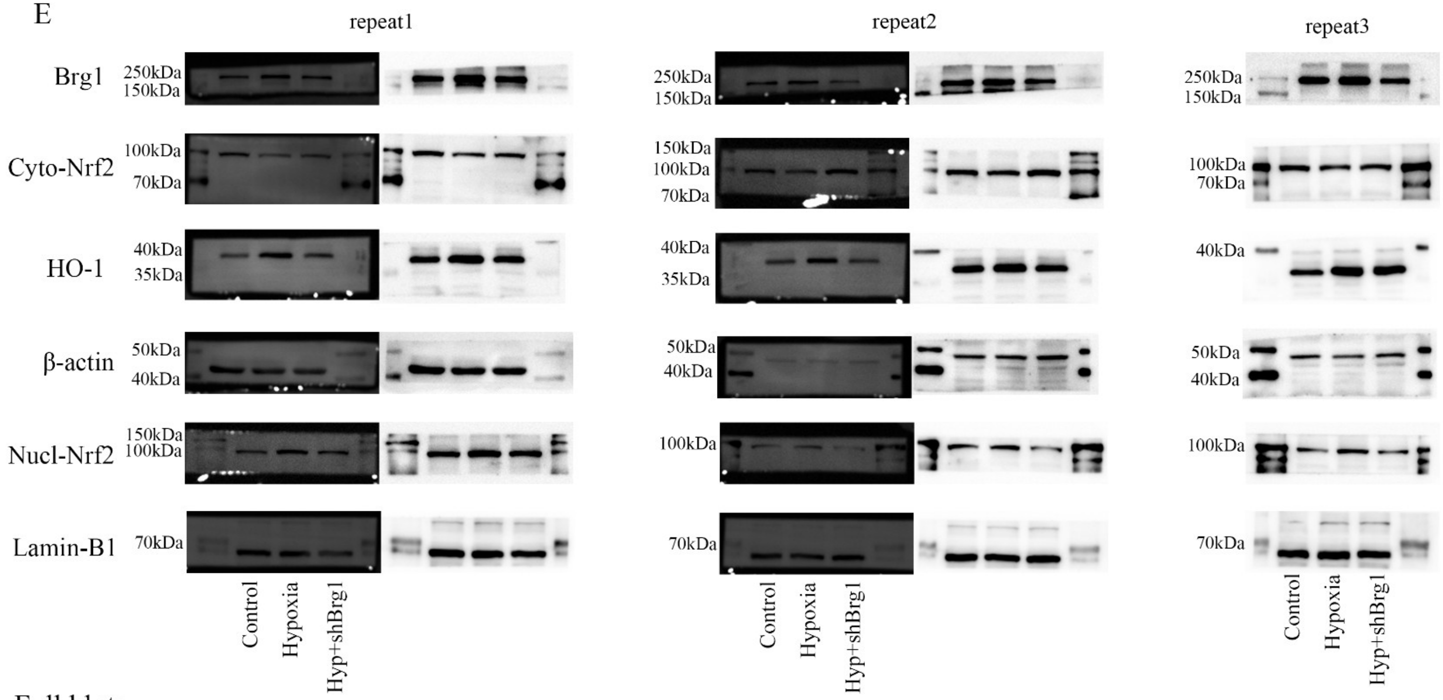

Full blots

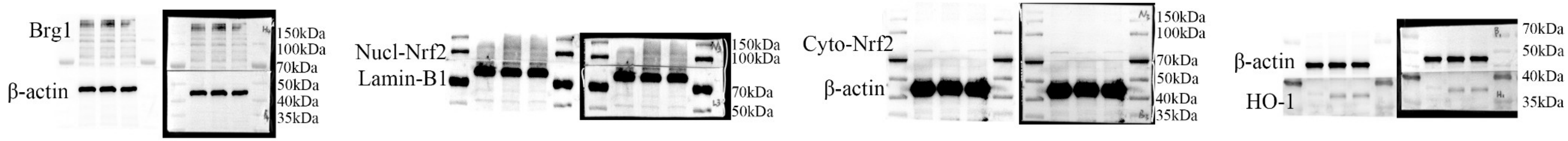

Figure10

C

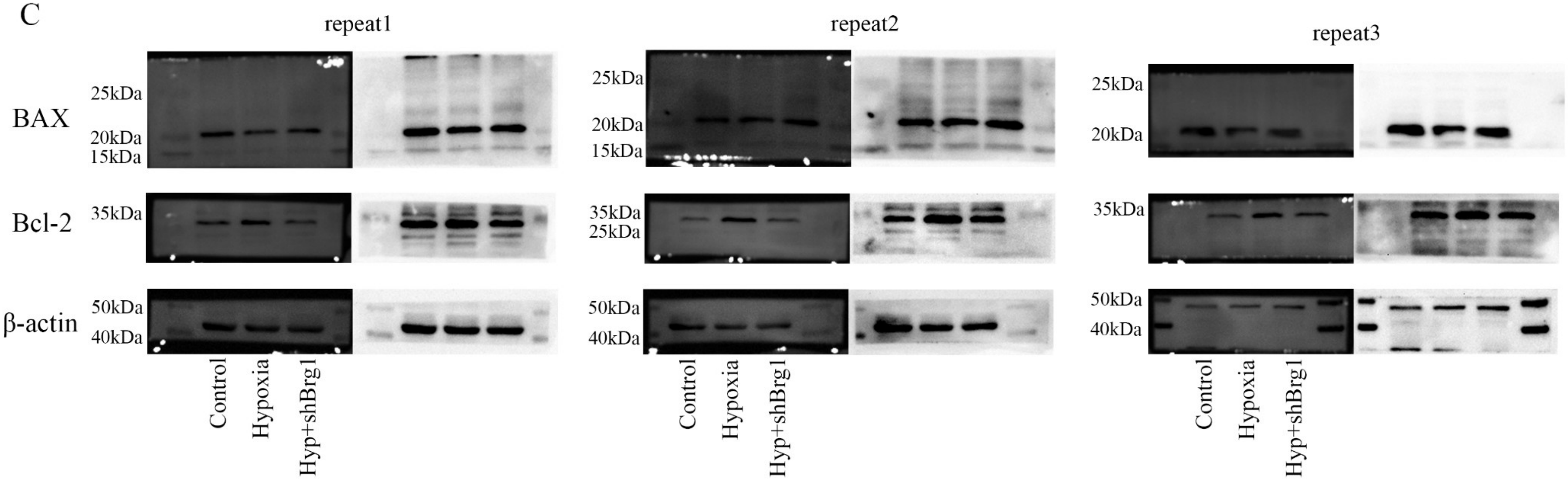

Full blots

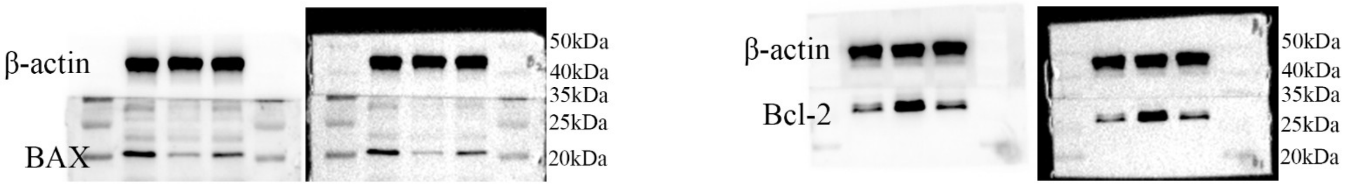

Figure11

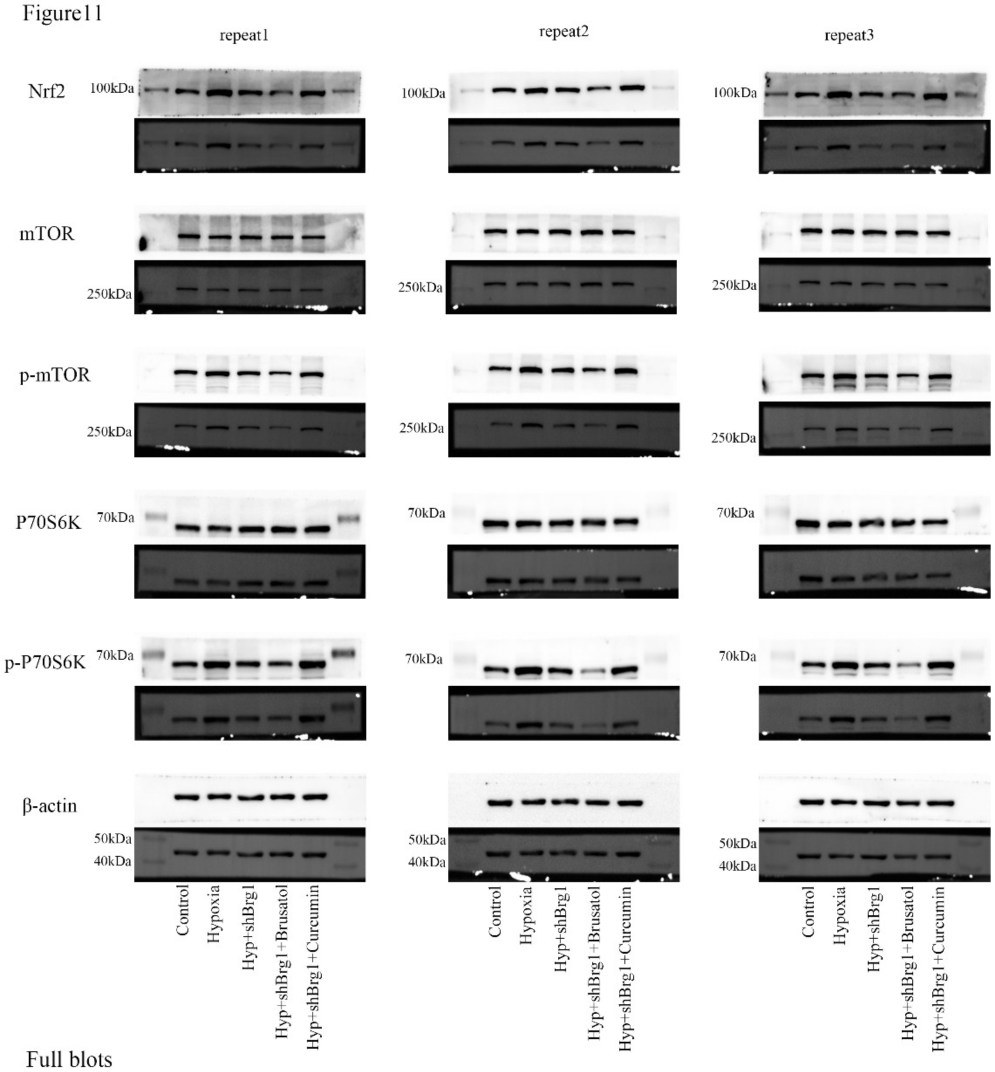

Full blots

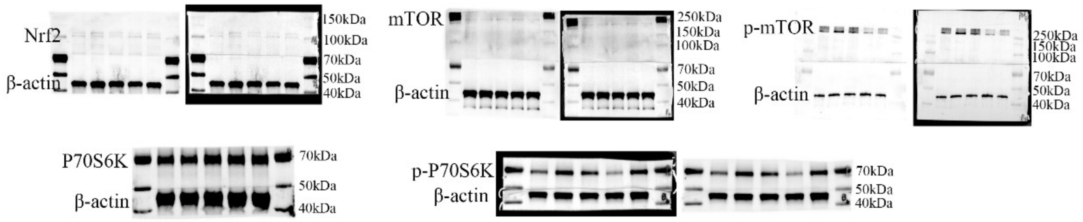

Supplement: Supplementary file 1 [file 41598_2026_55301_MOESM2_ESM.docx]
