# Supplementary material for: Loss of Brg1 prevents the progression of pulmonary hypertension by inhibiting Nrf2 expression
Source: Sci Rep. 2026 Jun 3;16:25395. doi: 10.1038/s41598-026-55301-6 (PMC13473476; doi:10.1038/s41598-026-55301-6)
Supplement: Supplementary file 1 [file 41598_2026_55301_MOESM1_ESM.docx]

**Supplementary File**

**Loss of Brg1 prevents the progression of pulmonary hypertension by inhibiting Nrf2 expression**

Wen-Di Jiang^1,2,3^, Qing Chen^1,3^, Mei-Yang Xu^1,3^, Guo-Qing Lu^3,4^, Zheng-Yu Sun^1,3^, Jia-Yi Guo^1,2^, Lei Wang^3,4^, Ying Qian^5^, Li Mu^5^, Yu-Hang Wang^6^, Ning-ning Zhang^3,4^, Hong-Ju Wang^3^, Bi Tang^3^, Pin-Fang Kang^3,4*^, Wen-Juan Wu^1,2^*

^1^ Bengbu Medical University Key Laboratory of Cancer Research and Clinical Laboratory Diagnosis, Bengbu Medical university, Bengbu, Anhui, 233000, P. R. China;

^2^ Department of Biochemistry and Molecular Biology, School of Laboratory Medicine, Bengbu Medical University, 233000, Bengbu, Anhui, P. R. China;

^3^ Department of Cardiovascular Medicine of The First Affiliated Hospital of Bengbu Medical University, Bengbu, Anhui, 233000; P. R. China;

^4^ Key Laboratory of Cancer Research and Clinical Laboratory Diagnosis, Bengbu Medical University; Bengbu, Anhui, 233000; P. R. China;

^5^ School of Laboratory Medicine, Bengbu Medical University, Bengbu, Anhui, 233000; P. R. China

^6^ School of Clinical Medicine of Bengbu Medical University, Bengbu, Anhui, 233000; P. R. China

^^^ These authors contributed equally to this work.

*Corresponding authors:

1. Pin-Fang Kang, Department of cardiovascular disease, the First Affiliated Hospital of Bengbu Medical College; 287 Chang Huai Avenue Bengbu 233004, China. Email: [kangpinfang.1016@163.com,](mailto:kangpinfang.1016@163.com(Pin-Fang) Tel: +86-571-3086107.
2. Wen Juan Wu, Department of School of Laboratory Medicine, 2600 Dong Hai Avenue Bengbu 233030, China, Email: [wuwj_2012@126.com](mailto:kangpinfang.1016@163.com(Pin-Fang), Tel: **+**86-571-3175909.

**Methods**

**ChIP-qPCR**

After the cells are intervened, prepare a sufficient amount of fresh cells. The number of cells per IP is approximately 4x106. Treat them with fresh 1% formaldehyde cross-linking, terminate the cross-linking with glycine, and collect the cells. Then, use a cell ultrasonic disruption instrument to disrupt the cells, breaking the genomic DNA into 100-500bp. Add 5μg of antibody and incubate with chromatin fragments overnight. Add protein A/G beads and incubate at 4℃ for 4-6 hours. Use Proteinase K to denature the cross-linking. Extract DNA with chloroform. Subsequently, use qRT-PCR to detect the binding situation of the target gene.

***Co-immunofluorescence staining***

Fixed lung tissue sections and treated PASMCs were perforated and membrane-broken in 0.3% Triton X-100 for 15 min at room temperature, washed with PBST, and then incubated with 1% bovine serum albumin (BSA) (Biosharp, China, BS114-250g) for 1 h. The sections and smooth muscle cells were then incubated with primary antibody at 4°C overnight. The next day, the sections and smooth muscle cells were rinsed three times with PBST and incubated for 2 h with secondary antibody. Cell nuclei were restained with DAPI Staining solution (Biosharp, China, BL105A). Images were captured with a fluorescence inverted microscope and analyzed with Image J software. Primary antibodies used were: Brg1 (Servicebio, China,GB11258), α-SMA (Servicebio, China, GB111364), CD31 (Servicebio, China,GB113151). Secondary antibodies used: Cy3 (ABclonal, China, AS007), Cy5 (Servicebio, China, GB27303).

Results

1.The shRNA used in this paper was provided by [WZ Biosciences Inc]. The sequence targeting rat Brg1 is as follows:

shRNA1: GGAAAGCGAGGAGGAAGAAGATTCAAGAGATCTTCTTCCTCCTCGCTTTCCTTTTTT,

shRNA2:

GGGCATATGAATTTGACAATTCAAGAGATTGTCAAATTCATATGCCCTTTTTT,

shRNA3:

GCTCTGTCAAGGTAAAGATCATTCAAGAGATGATCTTTACCTTGACAGAGCTTTTTT

shRNA4:

GCAAGGATTGGACCTATATTTCAAGAGAATATAGGTCCAATCCTTGCTTTTTT

**Figure S1. The binding situation of the Brg1 protein to the promoter region of the Nrf2 gene**

A. Schematic diagram of primer design, B. the results of qRT-PCR.

2. The results demonstrated that Brg1 co-localized primarily with α-SMA–positive cells, indicating that Brg1 is mainly expressed in PASMCs (Figure S2). These findings confirm the cell type-specific expression pattern of Brg1 in the pulmonary vasculature of PAH models.

**Figure S2 Co-immunofluorescence staining of Brg1, α-SMA and CD31 in animal model of PAH.**

A-B, Immunofluorescence staining and statistical analysis of Brg1 and CD31 in pulmonary arteries of Control and MCT groups. C-D, Immunofluorescence staining and statistical analysis of Brg1 and α-SMA in pulmonary arteries of Control and MCT groups.E-F, Immunofluorescence staining and statistical analysis of Brg1 and CD31 in pulmonary arteries of Control and Hypoxia groups.G-H, Immunofluorescence staining and statistical analysis of Brg1 and α-SMA in pulmonary arteries of Control and Hypoxia groups. Data was expressed as mean ± SD, n=3. *Compared with Control group, **P<0.01.
